# The ectomycorrhizal fungus *Paxillus involutus* converts organic matter in plant litter using a trimmed brown-rot mechanism involving Fenton chemistry

**DOI:** 10.1111/j.1462-2920.2012.02736.x

**Published:** 2012-06

**Authors:** Francois Rineau, Doris Roth, Firoz Shah, Mark Smits, Tomas Johansson, Björn Canbäck, Peter Bjarke Olsen, Per Persson, Morten Nedergaard Grell, Erika Lindquist, Igor V Grigoriev, Lene Lange, Anders Tunlid

**Affiliations:** 1 Department of Biology, Microbial Ecology Group, Ecology BuildingSE-22362 Lund, Sweden; 2 Department of Biotechnology and Chemistry, Aalborg UniversityLautrupvang 15, DK-2750, Ballerup, Denmark; 3 Centre for Environmental Sciences, Hasselt UniversityBuilding D, Agoralaan, 3590 Diepenbeek, Limburg, Belgium; 4 NovozymesKrogshoejvej 36, DK- 2880 Bagsvaerd, Denmark; 5 Department of Chemistry, Umeå UniversitySE-901 87 Umeå, Sweden; 6 US Department of Energy, Joint Genome Institute2800 Mitchell Avenue, Walnut Creek, CA94598, USA

## Abstract

Soils in boreal forests contain large stocks of carbon. Plants are the main source of this carbon through tissue residues and root exudates. A major part of the exudates are allocated to symbiotic ectomycorrhizal fungi. In return, the plant receives nutrients, in particular nitrogen from the mycorrhizal fungi. To capture the nitrogen, the fungi must at least partly disrupt the recalcitrant organic matter–protein complexes within which the nitrogen is embedded. This disruption process is poorly characterized. We used spectroscopic analyses and transcriptome profiling to examine the mechanism by which the ectomycorrhizal fungus *Paxillus involutus* degrades organic matter when acquiring nitrogen from plant litter. The fungus partially degraded polysaccharides and modified the structure of polyphenols. The observed chemical changes were consistent with a hydroxyl radical attack, involving Fenton chemistry similar to that of brown-rot fungi. The set of enzymes expressed by *Pa. involutus* during the degradation of the organic matter was similar to the set of enzymes involved in the oxidative degradation of wood by brown-rot fungi. However, *Pa. involutus* lacked transcripts encoding extracellular enzymes needed for metabolizing the released carbon. The saprotrophic activity has been reduced to a radical-based biodegradation system that can efficiently disrupt the organic matter–protein complexes and thereby mobilize the entrapped nutrients. We suggest that the released carbon then becomes available for further degradation and assimilation by commensal microbes, and that these activities have been lost in ectomycorrhizal fungi as an adaptation to symbiotic growth on host photosynthate. The interdependence of ectomycorrhizal symbionts and saprophytic microbes would provide a key link in the turnover of nutrients and carbon in forest ecosystems.

## Introduction

The total soil organic matter (SOM) corresponds to more than three times as much carbon (C) as that contained in the atmosphere or within terrestrial vegetation (Schmidt *et al*., 2011). A major part of this carbon occurs in forests (Falkowski *et al*., 2000). It was earlier believed that the C turnover of SOM is mainly controlled by the input of above-ground plant material and the decomposing activity of saprophytic organisms. However, an increasing amount of evidence now suggests that plant roots and their associated microbial communities play an important role in SOM dynamics (Read *et al*., 2004; Högberg and Read, 2006; Schmidt *et al*., 2011). The organisms that provide the largest sink for this C in boreal and temperate forests are the fungal root symbionts that form ectomycorrhizae (ECM). Estimates suggest that 10–50% of the C fixed by photosynthesis is allocated belowground to the ECM fungi (Simard *et al*., 2002). Other experiments show that up to one third of the soil microbial biomass and half of the dissolved organic carbon produced in forest soils originate from ECM symbionts (Högberg and Högberg, 2002).

The ECM mycelia prospect the soil for essential nutrients such as nitrogen (N) and transfer these to the host plant. A large portion of the soil N is present in an organic form including in particular proteins and amino acids (Nannipieri and Eldor, 2009). These N compounds are associated with polyphenols, polysaccharides and other degradation products of microbial and plant biopolymers that are present in SOM (Piccolo, 2001; Chengrong and Zhihong, 2008). The facts that polyphenols, such as tannins, humic acids and fulvic acids, can bind to, and form recalcitrant complexes with proteins limits their accessibility for the fungi (Bending and Read, 1996), suggesting that the saprotrophic activities of ECM fungi should be directed not only to organic nutrient compounds in soil, but also to other C-containing soil polymers. Several studies have demonstrated that at least some species of ECM fungi can decompose components of the major classes of organic compounds like proteins, pectins, cellulose, hemicelluloses and polyphenols commonly found in soils (Norkrans, 1950; Trojanowski *et al*., 1984; Haselwandter *et al*., 1990). Studies in soil microcosms have also shown that patches containing SOM are actively colonized by ECM fungi and depleted of their nutrients (Bending and Read, 1995a, b). More recently, it has been proposed by Talbot and colleagues (2008) that ECM fungi may live as facultative saprotrophs, i.e. they can degrade and metabolize soil C compounds as an alternative C source when the supplies of photosynthate from the host plants are low. Considering the large biomass of the ECM mycelia, this type of saprotrophic activity might represent a significant, but as yet largely unaccounted for, pathway of C loss in forest ecosystems.

However, the evidences that ECM fungi can act as decomposers have been questioned and the mechanisms by which ECM fungi may decompose organic compounds are poorly characterized. Analyses of the genomes of ECM fungi have shown that in contrast to saprophytic fungi ECM fungi have a reduced set of genes encoding plant cell wall-degrading enzymes (Martin *et al*., 2008; Nagendran *et al*., 2009). Furthermore, results from experiments examining the saprotrophic activity of ECM fungi in soil microcosms and field settings have been contradictory (Bending and Read, 1995a, b; Colpaert and Van Laere, 1996). It has also been argued that the assays commonly employed to measure the saprophytic activity of ECM fungi are unspecific and do not properly capture the decomposing activities (Baldrian, 2009). Other authors have suggested that because ECM fungi are confined to deeper soil horizons (Lindahl *et al*., 2007), which contain more decomposed litter and humus material of low energetic value, they are unlikely to be able to grow as facultative saprophytes on this material (Baldrian, 2009).

In the present study, we investigated the mechanisms by which the ECM fungus *Paxillus involutus* degrade complex organic matter extracted from plant litter material. *Pa. involutus* (Batsch) Fr. (*Basidiomycetes*; *Boletales*) is widely distributed in the Northern hemisphere, and is one of the best-studied ECM fungi, especially with respect to its ecology and physiology (Wallander and Söderström, 1999). Chemical modifications of the organic matter were analysed by spectroscopic methods. We find that during the assimilation of organic N, *Pa. involutus* modifies the major components of the organic matter by producing hydroxyl radicals through a Fenton system similar to that of saprophytic, wood decomposing brown-rot fungi (Green III and Highley, 1997). Transcriptome analyses showed that *Pa. involutus* expresses an extracellular enzyme system that mediates radical-based biodegradation of polymers contained in the organic matter, including polyphenols. However, the transcriptome lacked transcripts encoding enzymes needed for metabolizing the C liberated by depolymerization of soluble polysaccharides. We suggest that the liberated C is acquired by commensal microbes (such as soil-living, saprophytic bacteria and fungi) and that the ability to metabolize this C has been lost in ECM fungi as an adaptation to symbiotic growth on host photosynthate.

## Results

### Growth conditions and experiments

Three organic extracts were used: forest litter extracted with hot water (FH), and a maize compost extracted with cold (MC) or hot (MH) water. The extracts varied considerably in their content of C, N and other nutrients. During the 7 days of incubation, *Pa. involutus* assimilated between 33% and 53% of the total N present in the organic matter extracts. The added glucose was not detected at the end of the incubations (Table S1). At the end of the incubations, the pH of the FH medium decreased (from pH 4.0 to 3.7), while it increased in the MH medium (pH 5.5 at the end of incubation) and the MC medium (pH 5.4).

### Chemical conversion of organic matter

**Fig. 1:**
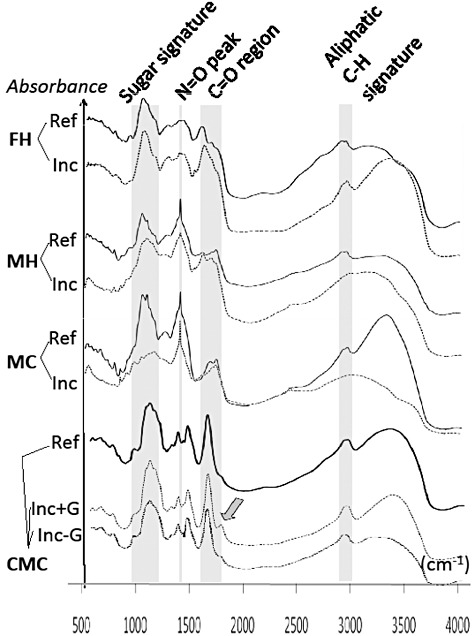
FTIR spectra of the three organic matter extracts and carboxy methyl cellulose (CMC) before (Ref, reference) and after 7 days of incubation (Inc, Inoculated) with *Pa. involutus*. FH, forest litter extracted with hot water; MH, maize compost extracted with hot water; MC, maize compost extracted with cold water. In the CMC spectra, the arrow indicates the appearance of a new peak located in the carbonyl region which is indicative of Fenton induced, oxidative modification of cellulose; +G and −G indicate CMC medium with or without supplement of glucose. Three replicates were analysed for the Inoculated and five for the Reference samples. The variation of the spectra between replicates was very low (Fig. S1).

Comparison of the FTIR spectra of the FH, MH and MC extracts before and after incubation showed that the chemical composition of the organic extracts changed during growth of the *Pa. involutus* mycelium. Pronounced differences were observed in four spectral regions characteristic of specific vibration modes*:* sugar modes (970–1200 cm^−1^), nitrate stretching modes (1350–1450 cm^−1^), C–O/C=O stretching modes (1500–1800 cm^−1^) and aliphatic C–H stretching modes (2850–3000 cm^−1^) (Fig. 1). In all inoculated extracts, several prominent peaks of the sugar region substantially decreased in intensity, which indicates changes in the relative abundance of sugar molecules including polysaccharides (Fig. S1). However, due to overlapping bands and spectral similarities, detailed assignment of the sugar composition based on infrared spectra is difficult and not very precise. The changes of the FITR spectra at 2850–3000 cm^−1^ showed that relative abundance of molecules containing aliphatic C–H groups were modified, which is consistent with the changes discussed in the sugar region. The spectral region containing C–O/C=O stretching modes differed among the organic matter extracts; still the effect of fungal incubation was evident for all three samples in this region as well. The spectral changes suggest that the relative concentrations of carboxyl, ketone, aldehyde and amide groups were altered.

**Fig. 2:**
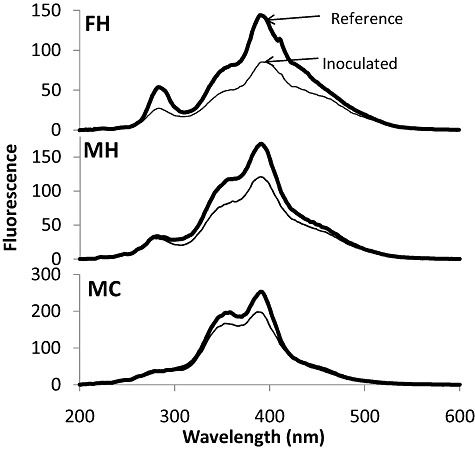
Synchronous fluorescence spectroscopy of the three organic matter extracts before (Reference, thick line) and after 7 days of incubation (Inoculated, thin line) with *Pa. involutus*. FH, forest litter extracted with hot water; MH, maize compost extracted with hot water; MC, maize compost extracted with cold water. Replicates (*n* = 3) of the samples were pooled before being analysed.

The synchronous fluorescence spectra of the FH, MH and MC extracts contained three prominent peaks: Peak 1, located around 280–300 nm, could be associated with monoaromatic rings; Peak 2 (330–360 nm), with more complex molecules with two condensed aromatic rings; and Peak 3 (360–400 nm) with more complex aromatic ring systems bearing carbonyl or carboxyl groups (Senesi *et al*., 1991) (Fig. 2; Table S2). In the FH extracts, the fluorescence intensity of all peaks decreased during incubation, which suggests that both simple and more complex aromatic compounds were at least partly degraded (decrease in concentration) or transformed (modification of the degree of polycondensation, amount of conjugated chromophores, or of the degree of electron-donating substitution) by *Pa. involutus*. In the MH and MC extracts, it was mainly the more complex aromatic compounds (Peak 3) that were degraded or transformed during the incubation.

**Fig. 3:**
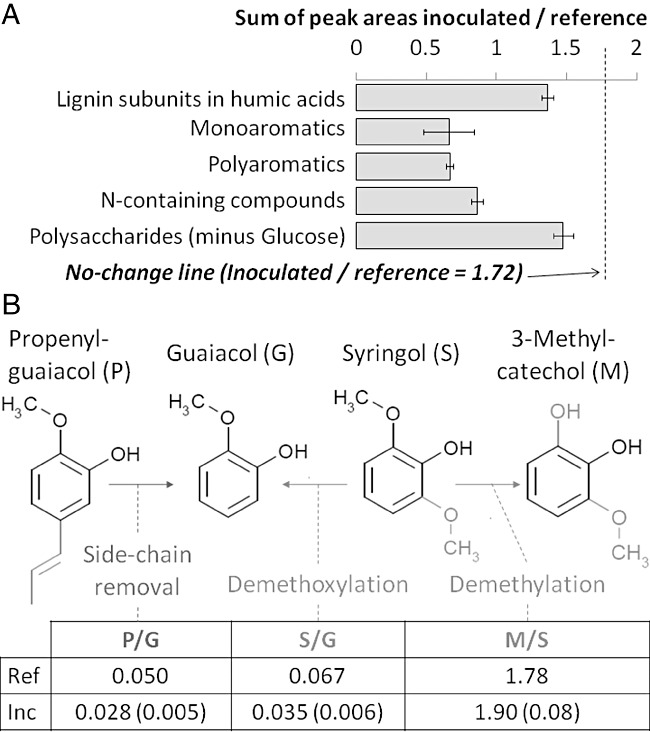
Pyrolysis GC/MS analysis of the organic matter extracted from forest litter using hot water (FH) after 7 days of incubation (Inoculated) and before incubation (Reference). A. Relative amounts of the major groups of organic compounds. A ratio below the ‘No change line’ indicates that this particular class of pyrolysis products was depleted in the Inoculated as compared with the Reference samples. ‘Lignin’ does not refer to genuine plant lignin but rather parts of the lignin molecule that are present in the humic acids as residuals of the degradation process. B. Chemical modification of lignin residuals subunits. Numbers indicate the relative peak area of the different lignin subunits in the reference sample against the average relative peak area in the incubated samples (*n* = 5, error bars denote standard error).

Analysis of the FH litter extract using Py-GC/MS confirmed that *Pa. involutus* degraded and converted the major classes of organic compounds present in dissolved organic matter (DOM) extracts (Fig. 3A; Table S3). The relative amount of pyrolysates related to aromatics, polyaromatics and N compounds decreased at least twice during the incubation. Relative amounts of pyrolysates issued from lignin subunits, present in the humic acids as residuals of the degradation process (Piccolo, 2001), and polysaccharides were less affected. However, detailed analysis of the pyrolysates related to lignin residuals showed that its two major subunits guaiacyl and syringyl were modified during growth of the fungus (Fig. 3B). The concentration of propenylguaiacol relative to guaiacol increased, indicating depletion of inter-unit ether linkages. The decrease in syringol/guaiacol ratio and the increase in 3-methoxycatechol pyroylsates relative to syringyl units showed that the syringyl units were both demethoxylated and demethylated.

Size-exclusion chromatography showed that a majority of the soluble aromatic compounds and carbohydrate containing molecules present in the three organic matter extracts were in the size range of ∼ 35–70 kDa (Fig. S2). We did not observe an alteration of the size distribution of the aromatic compounds in the incubated extracts; these polymers had the typical size (Piccolo, 2001) and synchronous fluorescence spectrum of humic acids (Ferrari and Mingazzini, 1995). In contrast, the size of the main peak of polysaccharides decreased during the incubation, which suggests that they were at least partially depolymerized during fungal growth.

We also examined whether *Pa. involutus* was able to chemically modify a cellulose substrate (carboxy methyl cellulose, CMC) and if this conversion was consistent with a mechanism involving free radical oxidation. The FTIR spectra of the substrate before and after 7 days of incubation were almost identical apart from the appearance of a new peak (λ∼ 1730 cm^−1^) located in the C=O stretching region (Fig. 1). Notably, such distinct increase in the intensity of carbonyl groups did only occur if the medium was supplemented with glucose (Fig. 1).

### Extracellular enzyme and iron reducing activities

When comparing the activity of extracellular enzymes in the organic matter media (FH, MH and MC) before and after 7 days of incubation, a significant increase in the activities of laccase and other oxidases was detected in two out of the three media (Fig. S3). In contrast, the activities of lignin peroxidase and cellulase were very low and most likely *Pa. involutus* did not secrete such enzyme activities during the degradation of DOM. Among hemicellulases, the activity of xylanase, but not β-glucuronidase, increased during the incubation.

A key requirement for the Fenton mechanism is a system for reduction of Fe^3+^ to Fe^2+^, which might be accomplished by extracellular fungal metabolites or reductive enzymes (Green III and Highley, 1997; Baldrian and Valaskova, 2008). Analysis of the iron-reducing activity in the organic matter extracts showed that it was significantly increased during decomposition (Fig. S4). Accordingly, iron-reducing compound(s) was formed during organic matter degradation.

### Secretome analysis

A cDNA library constructed from mycelia grown on the FH, MH and MC substrates was screened for transcripts encoding secreted or membrane bound proteins (Grell *et al*., 2011). In total, 18 contigs were identified that encode secreted enzymes with a possible role in organic matter degradation (Table S4). Five cDNA sequences were homologous to plant cell wall polysaccharide degrading enzymes. One sequence was predicted to encode an expansin family protein (TAST-1). Consistent with the enzymatic activity measurements, we did not identify any enzyme from the canonical crystalline cellulose decomposition system (GH6, 7 and 45) (Lynd *et al*., 2002). Instead, we found one endo-beta-1,4-glucanase of the GH9 family (TAST-2). However, the gene model did not bear a cellulose-binding module, and was therefore unlikely to bind and efficiently degrade crystalline cellulose (Kostylev *et al*., 2012). We identified three GH61 genes (TAST-5, 6, 7).

A number of oxidoreductases has been suggested to support Fenton chemistry, among them glyoxal oxidases that produces H_2_O_2_ (Baldrian and Valaskova, 2008). We identified a putative galactose oxidase/glyoxal oxidase among the TAST clones. Accumulation and regulation of oxalic acid secreted by the fungus is thought to be also involved in Fenton chemistry (Baldrian and Valaskova, 2008). TAST-13 is a predicted oxalate decarboxylase, the key enzyme in one of the two oxalate removal systems described in white-rot fungi (Mäkelä*et al*., 2010). Three TAST sequences were encoding enzymes potentially involved in decomposition of lignin residues: an aromatic peroxygenase (TAST-9), a cytochrome p450 oxidoreductase (TAST-10) and a laccase (TAST-11).

### Transcriptome profiling

**Fig. 4:**
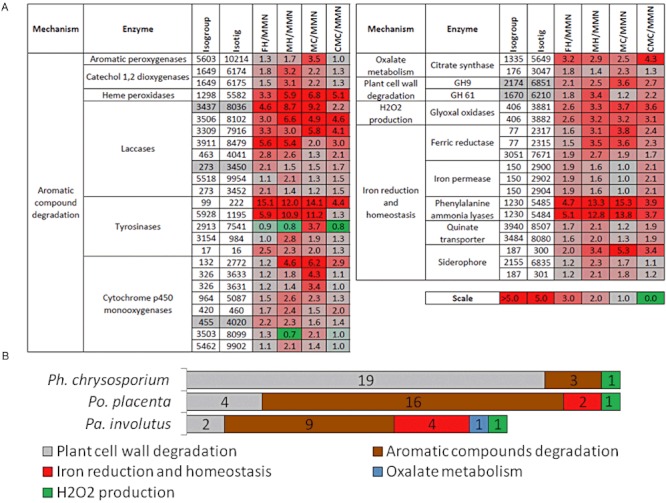
Regulation of genes potentially involved in organic matter degradation by *Pa. involutus*. A. Expression profile of 44 genes that were manually annotated as potentially involved in organic matter degradation, and were upregulated more than twice (false discovery rate q < 0.01) in at least one of pairwise comparisons in media containing extracts of complex organic material versus mineral nutrient medium (MMN). The data presented are average ratio of expression (*n* = 3). Four different types of organic substrates were used: forest litter extracted with hot water (FH), maize compost extracted with hot water (MH), maize compost extracted with cold water (MC) and carboxy methyl cellulose (CMC). Isotigs and isogroups refer to transcripts and genes respectively. In grey boxes are 5 isotigs that were also identified in the TAST screening (Table S4). B. Comparison of the transcriptional response of *Po. placenta, Ph. chrysosporium* and *Pa. involutus* when growing on a cellulose medium as compared with a medium containing glucose as the carbon source. The number of genes that were upregulated at least twofold (in average of three replicates) and those with annotations consistent with a potential role in organic matter degradation are shown. Microarray data for *Ph. chrysosporium* and *Po. placenta* growing on microcrystalline cellulose (AVICEL) and glucose media were downloaded from the GEO database (accession numbers GSE14736 and GSE12540 respectively).

Microarray analysis showed that the transcriptome profiles of the mycelium grown on the three organic extracts were similar but distinctly different from the ones grown on the MMN medium (Fig. S5). In total, 73 isotigs (transcripts) that encoded putative organic matter degrading enzymes and proteins were significantly upregulated more than twofold in at least one of the pairwise comparisons FH/MMN, MH/MMN and MC/MMN (Fig. 4A). The 73 isotigs belonged to 60 isogroups (genes). The regulations of the isotigs from the same isogroup were almost identical.

Twenty-seven of the 60 upregulated genes were predicted to encode Carbohydrate-Active enzymes (CAZymes). However, only two of them, a GH9 (endoglucanase) and a GH61, were expected to be involved in plant cell wall degradation. The remaining CAZymes most likely have a role either in the synthesis and turnover of the fungal cell wall, the degradation of chitin-containing compounds or in glycoprotein modification (not shown in Fig. 4A). Twenty-two genes were induced encoding enzymes possibly involved in lignin degradation, such as multicopper oxidases, p450 oxidases, tyrosinases and catechol dioxygenase. Nine of the upregulated genes were found to encode for iron metabolism-related proteins and were therefore potentially involved in Fenton reaction. We also found two genes coding for proteins involved in oxalate/citrate metabolism (citrate synthase) and one coding for a glyoxal oxidase.

The transcriptional response of the fungus when grown on cellulose (CMC) was similar to the response when grown on the FH, MH and MC organic matter extracts (Fig. 4A; Fig. S5). In all media, at least one transcript was significantly upregulated among the glyoxal oxidases, phenylalanine ammonia lyases, haem peroxidases, laccases, tyrosinases, cytochrome p450 oxidoreductases, citrate synthases and endoglucanases (GH9) respectively. However, the expression levels of specific transcripts within these families differed depending on the substrate.

### Comparative transcriptome analysis

Finally, we compared the set of genes that were significantly upregulated during conversion of cellulose in the ECM fungus *Pa. involutus,* the white-rot fungus *Phanerochaete chrysosporium* and the brown-rot fungus *Postia placenta* (Fig. 4B). The cohort of overexpressed genes of *Ph. chrysosporium* was dominated by plant cell-wall degrading CAZymes. Moreover, *Ph. chrysosporium* lacked transcripts encoding proteins involved in pigment production, oxalic acid metabolism and iron metabolism. Such transcripts were together with oxidases involved in lignin degradation, prevalent in the upregulated transcriptomes of both *Po. placenta* and *Pa. involutus*. Six plant cell-wall degrading CAZymes were upregulated in *Po. placenta* including two GH1 (β-glucosidases), two GH3 (one β-glucosidase and one β-xylosidase), one GH 10 (endoxylanase/hemicellulase) and one GH28 (polygalacturonase). None of these CAZymes were upregulated in *Pa. involutus*. In this fungus two plant cell-wall degrading CAZymes were induced namely a GH9 and a GH61 (Fig. S6).

## Discussion

During the assimilation of organic N from the soluble organic material, *Pa. involutus* degraded at least part of the polysaccharides, while the polyphenols were not digested, even though some of their aromatic constituents were chemically modified. Such a selective depolymerization of polysaccharides is very similar to a brown-rot decay mechanism where cellulose and hemicelluloses (polysaccharides) are depolymerized and lignin (aromatic polymer) is not, but is still chemically modified (Green III and Highley, 1997). The pyrolysis analysis showed that the lignin subunits present in the polyphenols were modified by *Pa. involutus* through a significant reduction in the number of side-chains. Depletion of original C3 side-chains indicates oxidation of these groups during incubation, and is a good indicator of the degradation of lignin residues (Nierop *et al*., 2005). Demethylation of methoxyl groups linked to syringyl subunits, similar to our findings, has recently been detected in *in situ* analyses of brown-rotted wood by 2D NMR (Martinez *et al*., 2011) and has also been found to be consistent with an oxidative degradation of the lignin through Fenton reaction by the brown-rot fungus *Po. placenta* (Yelle *et al*., 2011). Likewise, the introduction of a sharp carbonyl-groups signal in the FTIR spectrum of cellulose, as observed when *Pa. involutus* was grown on CMC, has been associated with a Fenton reaction mechanism (Green III and Highley, 1997).

The Fenton reaction requires the reduction of Fe^3+^ to Fe^2+^ and iron reducing compounds were produced during the degradation of plant litter by *Pa. involutus*. Three mechanisms have been proposed for ferric reduction in basidiomycetes: (i) iron-reducing enzymes (ferric reductases, cellobiose oxidases, cellobiose dehydrogenase) (Baldrian and Valaskova, 2008), (ii) low-molecular-weight (LMW) glycopeptides (Vanden Wymelenberg *et al*., 2010), and (iii) redox cycling by small-molecular mass compounds like dimethoxyhydro- and benzoquinones: DMHQ, DMBQ (Newcombe *et al*., 2002). In support for the first mechanism, we identified two genes encoding putative ferric reductases that were significantly upregulated during the degradation of organic matter. However, we did not recognize any transcripts displaying sequence similarity to cellobiose dehydrogenase. This enzyme is also absent in the brown-rot fungus *Po. placenta* (Martinez *et al*., 2009). We identified three transcripts encoding proteins with sequence similarity to LMW iron reducing glycoproteins in *Ph. chrysosporium* (Tanaka *et al*., 2007). However, none of them were upregulated during organic matter degradation. The induction of genes involved in pigment synthesis (phenylalanine ammonia lyases and tyrosinases) and quinate transport suggests the involvement of a quinone redox-cycling iron reduction mechanism (Martinez *et al*., 2009; Vanden Wymelenberg *et al*., 2010). *Pa. involutus* is known to produce the pigment involutin (Feling *et al*., 2001): this molecule bear a catechol group that could undergo oxidation in the corresponding quinone, which could then be reduced again intracellularly after transport through the quinate transporter (see Fig. S7). However, the lack of electron-donating methoxyl groups (that are present in characterized hydroquinones that contribute to Fenton reaction, see Suzuki *et al*., 2006) makes this molecule more of a free radical scavenger rather than an iron reducing compound. We therefore hypothesize that the pigment is secreted by the fungus as a protection against its own oxidative machinery.

When degrading organic matter, *Pa. involutus* overexpressed a number of transcripts of oxidases like laccases, catechol dioxygenase, haem peroxidase, tyrosinases and cytochrome p450 monooxygenases that have also been showed to be produced by the brown-rot fungus *Po. placenta* when grown on wood or cellulose media (Martinez *et al*., 2009; Vanden Wymelenberg *et al*., 2010). No transcripts encoding class II peroxidases (Mn and lignin peroxidases) that are signatures for a white-rot mechanism were detected in *Pa. involutus*. Considering the expression of CAZymes involved in the degradation of plant cell walls, there were large differences between *Pa. involutus* and *Po. placenta* (see Fig. S6). Although, none of them expressed genes coding for enzymes of the canonical crystalline cellulose decomposition system (Lynd *et al*., 2002), the transcript profile of *Po. placenta* employs an array of CAZymes like endoglucanases, β-glucosidases and hemicellulase when it is grown on cellulose or aspen (Martinez *et al*., 2009; Vanden Wymelenberg *et al*., 2010). Except for one endoglucanase (GH9), no such glycosyl hydrolases were induced in *Pa. involutus* during growth on cellulose or plant litter. In addition to the GH9, a member of the GH61 family was significantly upregulated in *Pa. involutus*. GH61 is the most abundant CAZyme family acting on plant cell walls in the genome of *L. bicolor* (Martin *et al*., 2008). Recently, it has been reported that GH61 can depolymerize cellulose oxidatively in cooperation with cellobiose dehydrogenase or LMW reducing agents (Langston *et al*., 2011; Quinlan *et al*., 2011). Hence, GH61 could be an important component of the radical-based cellulose degrading mechanism of ECM fungi.

In saprophytic brown-rot fungi, the radical-based and enzymatic parts of the lignocellulose degrading system act synergistically (Baldrian and Valaskova, 2008; Yelle *et al*., 2011). Most likely, the capacity of *Pa. involutus* to express the enzymatic part has been lost during the evolutionary transition from a saprophytic brown-rot precursor (Binder and Hibbett, 2006) to a symbiotic ECM fungus relying on the plant host for carbohydrate provision. The genomic mechanisms that could account for this loss are not known. However, considering previous genome studies of ECM fungi showing a large reduction in the number of plant cell wall degrading enzymes (Martin *et al*., 2008; Nagendran *et al*., 2009), gene loss and deletions have likely played an important role. Moreover, in contrast to the situation in brown-rot fungi (Martinez *et al*., 2009), the polysaccharides present in the organic matter, even though depolymerized, were not assimilated. This was also consistent with the absence of gene models coding for cellobiohydrolase, the enzyme cleaving cellobiose into two glucoses, acting in the last step of cellulose degradation. Moreover, in contrast to the situation in brown-rot fungi (Martinez *et al*., 2009), oxidative degradation of cellulose did not occur in *Pa. involutus* without adding glucose to the medium. This observation suggests that mutations affecting transcriptional regulation have also contributed to the symbiotic adaptation of the brown-rot decay system.

The decomposing activities of ECM fungi in soils are commonly analysed using enzyme assays that include plant cell wall degrading enzymes (Courty *et al*., 2005). Considering the fact that such enzymes were not expressed during the degradation of plant litter material by *Pa. involutus*, and that very little enzymatic activities were measured in the organic matter extracts, measuring the activity of those enzymes might give misleading results. Laccases are also used as enzyme marker for saprophytic ECM activity (Courty *et al*., 2009). Although being upregulated by *Pa. involutus* during the decay of litter material, laccases are found in multigene families having members with complex patterns of expression. Hence, further studies are needed to develop molecular markers that can correctly capture the saprotrophic activity of ECM fungi in the field.

That ECM fungi do not assimilate C from decomposed plant litter has been indicated in field experiments using ^14^C-labelled litter material (Treseder *et al*., 2006). Several studies have also shown that the ECM mycelia are surrounded by distinct communities of saprophytic bacteria and fungi (Boer *et al*., 2005; Izumi and Finlay, 2011). Most likely, these communities contain commensals that grow on the C resources that become available during the radical-based degradation by the ECM fungi. Some of these microbes may strive for the same nutrient resources as the ECM fungi. Thus, it can be expected that ECM fungi have evolved mechanisms including the secretion of toxic metabolites that could control the activity of saprophytic microorganisms (Boer *et al*., 2005). Indeed, studies in soil microcosms have shown that the ECM mycelium of *Pa. involutus* can reduce the activity of saprophytic bacteria (Olsson *et al*., 1996). The combined metabolic activity of symbiotic fungi and saprophytic microbes may have a significant impact on the turnover of carbon and nutrients in forest soils. Moreover, being supplied by energy from the plant, this pathway could operate at deeper soil horizons that are energetically unavailable for traditional saprophytes (Lindahl *et al*., 2007).

A caveat of the present study is that the experiments were done using organic litter material in solution, i.e. the DOM fraction. Dissolved organic matter represents only a small proportion of the total SOM and lacks insoluble components like lignin, cellulose and chitin as well as aggregates formed between organic compounds and minerals (Bolan *et al*., 2011). It is well known that mineral particles could stabilize organic compounds against degradation by microbial enzymes (Allison, 2006). It remains to be determined to what extent the radical-based biodegradation system of ECM fungi can act on intact SOM material.

## Experimental procedures

### Fungal strain and culture conditions

Cultures of *Pa. involutus* (Batsch) Fr. (strain ATCC 200175) were maintained aseptically on 1.5% agar plates containing minimum Melin-Norkrans medium (MMN) (composition: 2.5 g l^−1^ glucose, 500 mg l^−1^ KH_2_PO_4_, 200 mg l^−1^ NH_4_Cl, 150 mg l^−1^ MgSO_4_·7H_2_O, 25 mg l^−1^ NaCl, 50 mg l^−1^ CaCl_2_, 12 mg l^−1^ FeCl_3_·6H_2_O, and 1 mg l^−1^ Thiamine-HCl; pH 4.0). The fungus was grown in Petri dishes on a layer of glass beads immersed in liquid medium (van Schöll *et al*., 1996). A monolayer of autoclaved 4 mm diameter glass beads was poured into the bottom of a 9 cm Petri dish and 10 ml of the MMN medium was added. A mycelial plug (*c.* 5 mm in diameter) was cut from the margin of an actively growing mycelium (MMN agar medium) and transferred to the centre of the glass bead plate. After 9 days of incubation (18°C, dark) when the diameter of the colony reached a size of approximately 4 cm in diameter and a biomass of 10 mg (dry weight), the MMN medium was removed. The glass beads and the mycelium were washed with 10 ml of sterile MilliQ (MQ) water, and 10 ml of MMN medium without N was added to induce a N-deprived mycelium. After 24 h, the mycelium was washed with MQ water and extracts of organic matter (10 ml) were added. In addition, the fungus was grown in CMC (10 g l^−1^). The organic matter extracts and the CMC medium were supplemented with glucose (final concentration 2.5 g l^−1^) before inoculation, to avoid a situation of carbon limitation. The cultures were incubated for 7 days at 18°C in the dark, just before the mycelium started being space-limited in the Petri dish.

### Preparation of organic matter extracts

Forest litter material was collected from the upper 10 cm soil layer in a 61-year-old pure spruce stand growing in N-poor site in central Sweden (soil pH = 5.0). The maize compost was produced by cutting maize leaves into small pieces and composting them in an isolated plastic compost bin for 12 months. The litter and compost material was extracted with either cold or hot water (Davidson *et al*., 1987). Three organic extracts were generated: forest litter extracted with hot water (FH), and a maize compost extracted with cold (MC) or hot (MH) water. Particles were removed by sequentially filtering (0.2 µm) and LMW compounds by ultra-filtration (cut-off 10 kDa). Further details are given in Supporting Information (Appendix S1).

### Chemical analysis

Samples for Fourier transform infrared (FTIR) spectroscopy were prepared by drying (vacuum over night at 4°C) 5 ml of the organic matter extracts. The FTIR spectrum was recorded using a Bruker IFS66 v/s spectrometer. Data were collected in diffuse reflectance mode using a praying mantis diffuse reflectance attachment (Harrick Sci.). Each spectrum was the result of 1000 consecutive scans at a resolution of 4 cm^−1^. Synchronous fluorescence spectra were obtained using a Perkin-Elmer LS50B fluorescence spectrophotometer. Samples (750 µl) were kept at room temperature (20°C) and processed at a 10 nm bandwidth and 25 nm offset (Δλ = 25 nm) between excitation and emission. Pyrolysis gas chromatography-mass spectrometry (Py-GC/MS) was performed using a Perkin Elmer TurboMass/ Autosystem XL with Frontier Lab double Shot pyrolyser. Size-exclusion chromatography was performed using a HiLoad 16/60 Superdex200 column (GE Healthcare). Further details are given in Supporting Information (Appendix S1).

### Enzyme activity measurements and ferrozine assay

To remove interfering compounds, the extracts were treated with PVPP (PolyVinyl Poly Pyrrolidone) (Pierpoint, 1996), followed by acetone precipitation. Laccase activity was measured using syringaldazine as a substrate (Leonowicz and Grzywnowicz, 1981), lignin peroxidase using veratryl alcohol (Tien and Kirk, 1988), overall oxidase activity using ABTS (Palmieri *et al*., 1997), cellobiohydrolase activity using methylumbelliferyl-β-d-cellobioside (Courty *et al*., 2005), glucuronidase activity using methylumbelliferyl-β-d-glucuronide hydrate (Courty *et al*., 2005), and xylanase activity using RBB-Xylan as substrate (Biely *et al*., 1985). The capacity of *Pa. involutus* to produce iron-reducing compounds was examined using a ferrozine assay (Goodell *et al*., 2006). Further details are given in Supporting Information (Appendix S1).

### Construction and screening of TAST cDNA library

Total RNA was isolated using the RNeasy Plant Mini Kit (Qiagen). A cDNA library was generated from RNA isolated of mycelia grown on the FH, MH and MC substrates. Poly(A) RNA isolation, cDNA synthesis, and transposon assisted signal trapping (TAST) cDNA library construction were essentially carried out as described previously (Grell *et al*., 2011). A collection of 576 TAST clones were selected for sequencing. The sequences were assembled into 348 contigs which were manually annotated. Further details are given in Supporting Information (Appendix S1).

### Transcriptome sequencing and microarray

The transcriptomes expressed by the fungus during growth on the FH, MH and MC extracts and the MMN medium, respectively, was sequenced using the 454 technology. In total, the sequencing yielded 2 029 605 reads that were assembled into a set of 12 873 isotigs. These isotigs represent various splice variants and they were mapped to 8620 genes or isogroups. Sequences may be accessed from http://mbio-serv2.mbioekol.lu.se/Paxillus/Hybrid/ (add ‘paxillus_’ to the given isotig and isogroup numbers). EST sequences are also available at GenBank SRA046093. Based on manual annotations, we identified 269 transcripts among the 12 873 isotigs that encodes for enzymes and proteins with a possible role in the degradation of organic matter (polysaccharide modifications, lignin degradation, iron reduction and homeostasis, oxalate metabolism and H_2_O_2_ production). Roche NimbleGen arrays were designed to assess expression of 12 214 isotigs. The data deposited in NCBI's Gene Expression Omnibus (Edgar *et al*., 2002) and are accessible through GEO Series accession number GSE34402 (http://www.ncbi.nlm.nih.gov/geo/query/acc.cgi?acc=GSE34402). Further details are given in Supporting Information (Appendix S1).

## Supporting information

**Fig. S1.** FTIR spectra of the sugar region.

**Fig. S2.** Size-fractionation of the organic matter extracts.

**Fig. S3.** Extracellular enzyme activities secreted by *Pa. involutus.*

**Fig. S4.** Iron-reducing capabilities in the organic extracts degraded by *Pa. involutus.*

**Fig. S5.** Principal component analysis of transcriptome data.

**Fig. S6.** Comparison of CAZYme repertoires of*Pa. involutus*, *Po. placenta* and *Ph. chrysosporium* when growing on cellulose.

**Fig. S7.** Proposed contribution of involutin in a hydroquinone-mediated Fenton reaction.

**Table S1.** Changes in C, N and nutrients during the conversion of soil organic matter.

**Table S2.** Data used for interpreting peaks in the synchronous fluorescence (SF) spectra.

**Table S3.** Putative identification of the pyrolysis compounds.

**Table S4.** Identification of secreted proteins using the TAST method.

**Appendix S1.** Supplementary experimental procedures.
