# Supplementary material for: The ectomycorrhizal fungus Paxillus involutus converts organic matter in plant litter using a trimmed brown-rot mechanism involving Fenton chemistry
Source: Environ Microbiol. 2012 Jun;14(6):1477–87. doi: 10.1111/j.1462-2920.2012.02736.x (PMC3440587; doi:10.1111/j.1462-2920.2012.02736.x)
Supplement: Supplementary file 1 [file emi0014-1477-SD1.pdf]

Figure S1. FTIR spectra of the sugar region of the three organic matter extracts before (Reference) and after 7 days of incubation (Inoculated) with *Pa. involutus* (the whole spectra are shown in Figure 1).The thick line is the average spectrum (three replicates for reference samples, five replicates for incubated ones) while the upper and lower thin lines represent the standard deviation. The wavelength ($cm^{-1}$) is in x-axis and the absorbance units are in y-axis. The main peaks in each spectrum have been identified and assigned to potential sugars according to Kacurakowa et al. (Kacurakova *et al.*, 2000).

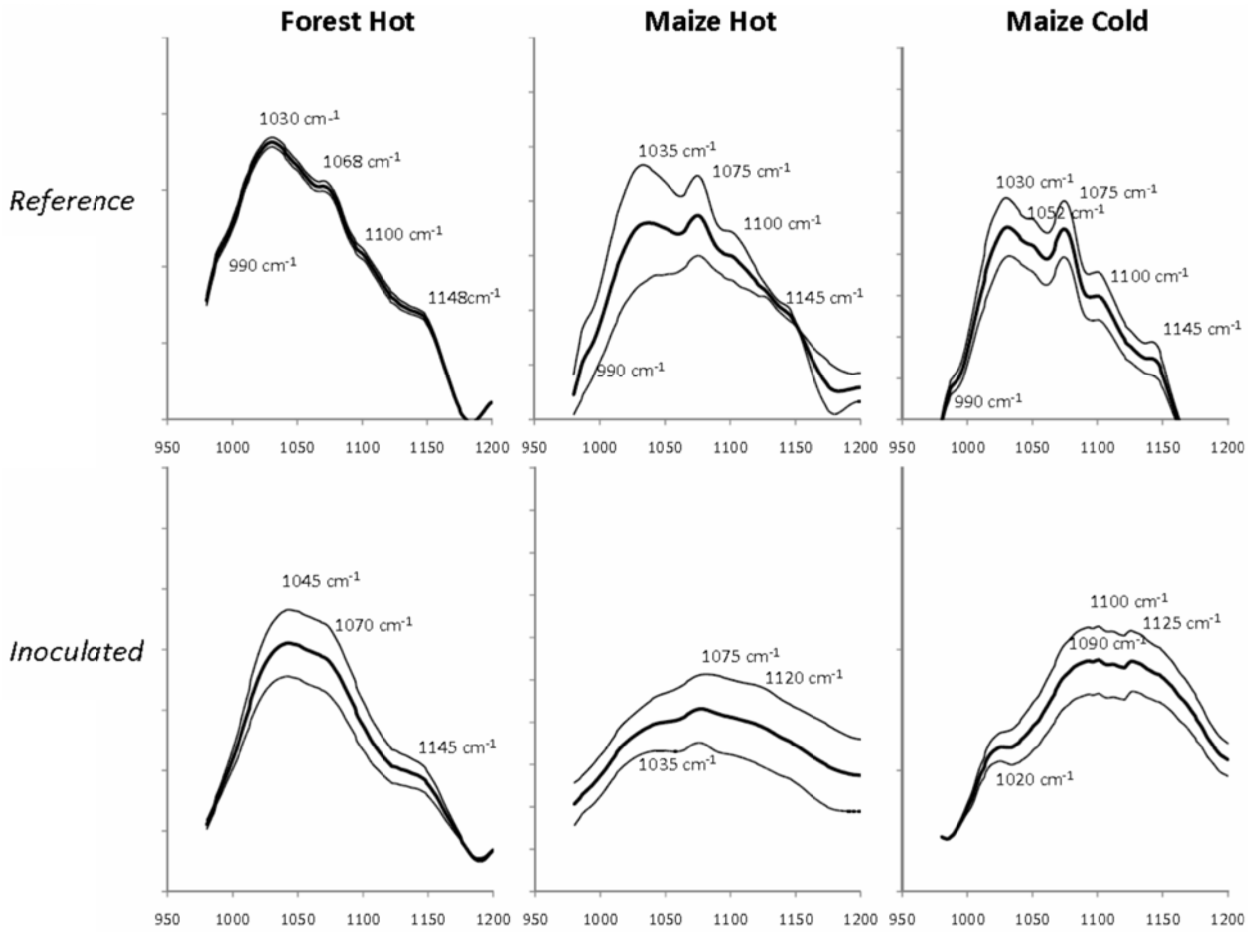

Figure S2. Size-fractionation of the organic matter extracts before (Reference, solid line) and after 7 days of incubation (Inoculated, dashed line) with *Pa. involutus*. A Superdex 200 column was used and the samples were eluted at a flow rate of 1 ml/min. Fractions (8ml) were collected and assayed for reducing sugars and aromatic compounds (synchronous fluorescence spectroscopy 400 nm peak).

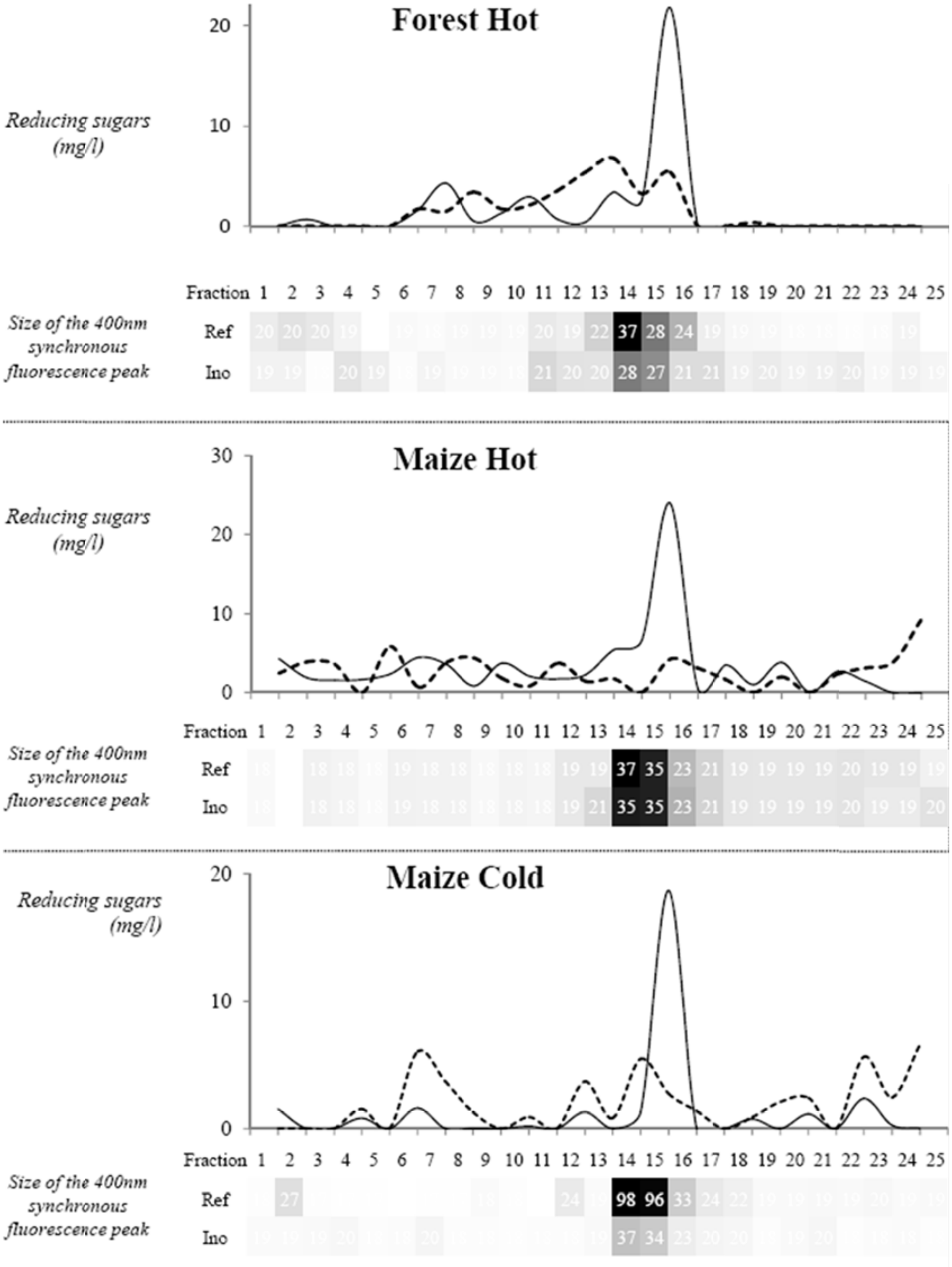

Figure S3. Extracellular enzyme activities secreted by *Pa. involutus* during growth on extracts of organic matter. Shown is the difference in enzyme activities measured in the extracts inoculated for 7 days and 0 days (i.e. the Reference). Bars indicate ±SEM (N=3).

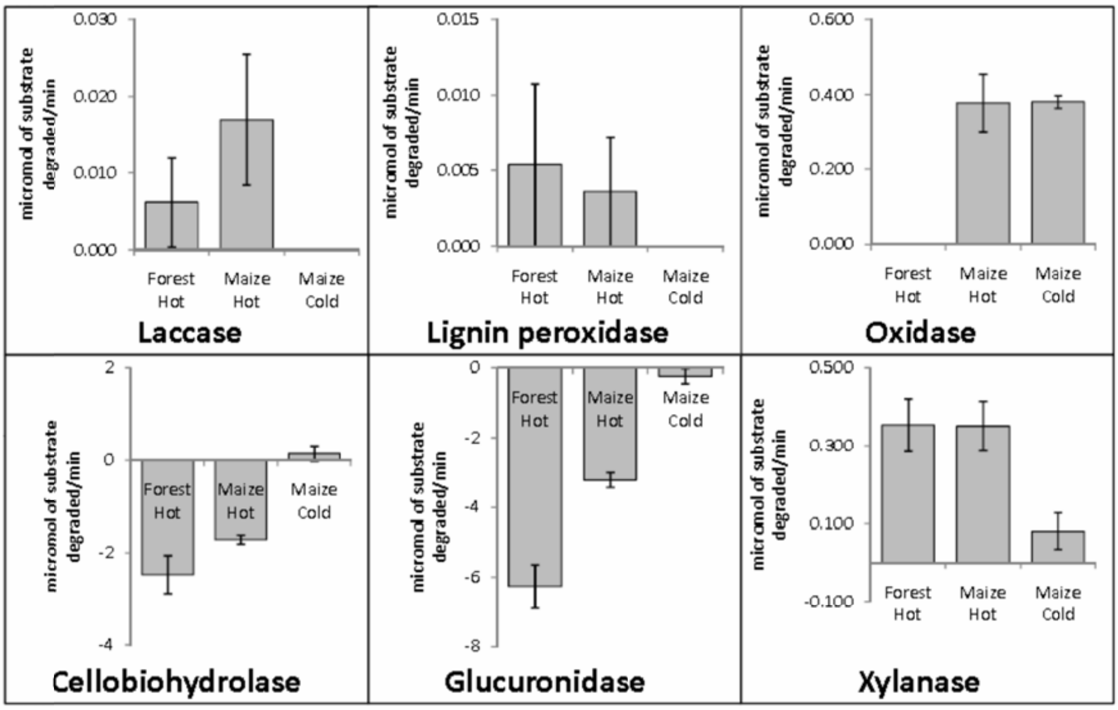

Figure S4. Iron-reducing capabilities in organic extracts degraded by *Pa. involutus* after 2, 4 and 6 days of growth. Reference is an extract before inoculation of the fungus. Bars indicate ± 1 SEM (N=5).

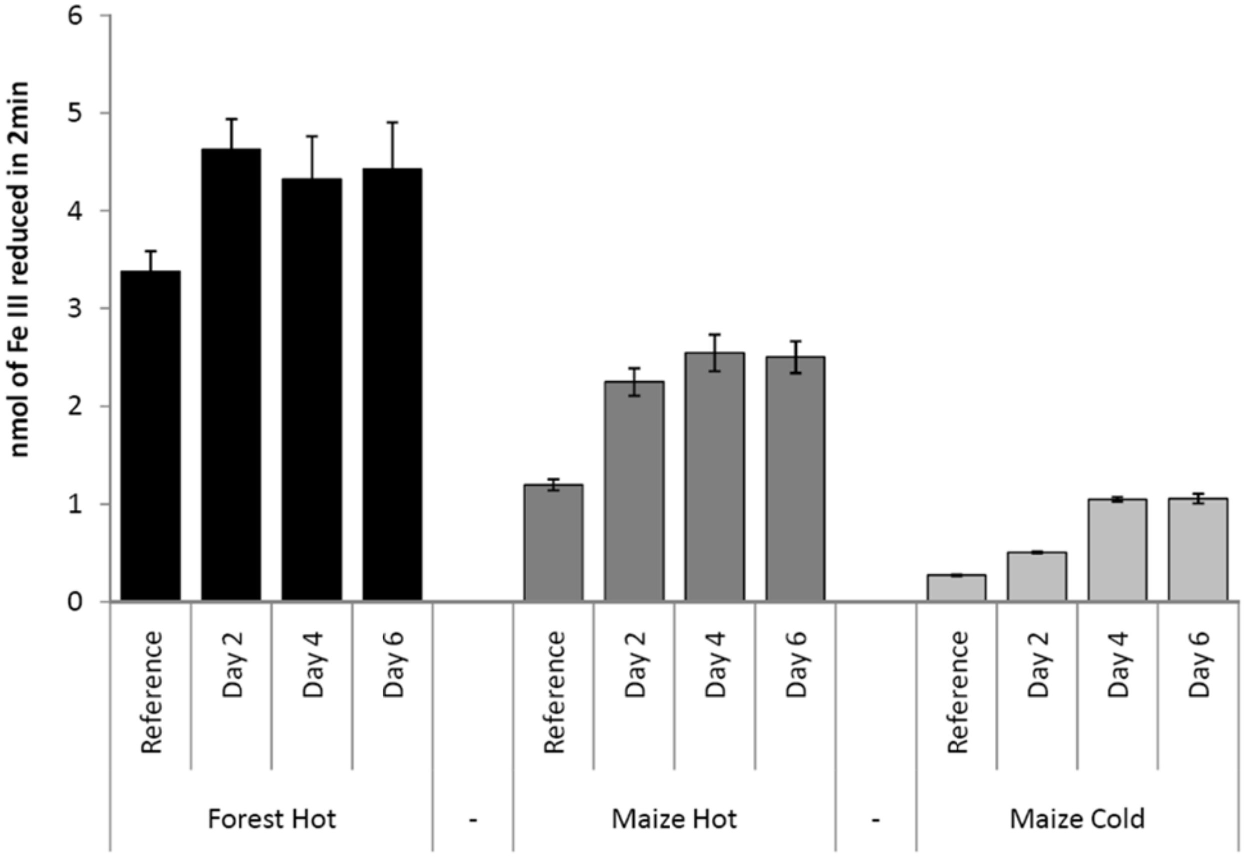

Figure S5. Principal component analysis of the expression levels of the 5,556 gene models (out of 12,214) that had a false discovery rate of q<0.01. MMN: Minimum Melin Norkrans medium, CMC: Carboxy Methyl Cellulose, FH, forest litter extracted with hot water; MH, maize compost extracted with hot water; MC, maize compost extracted with cold water. Each sample (pooled mycelium from three Petri dishes) has been run in triplicates, except for MMN (n=6); each point in the PCA is a replicate.

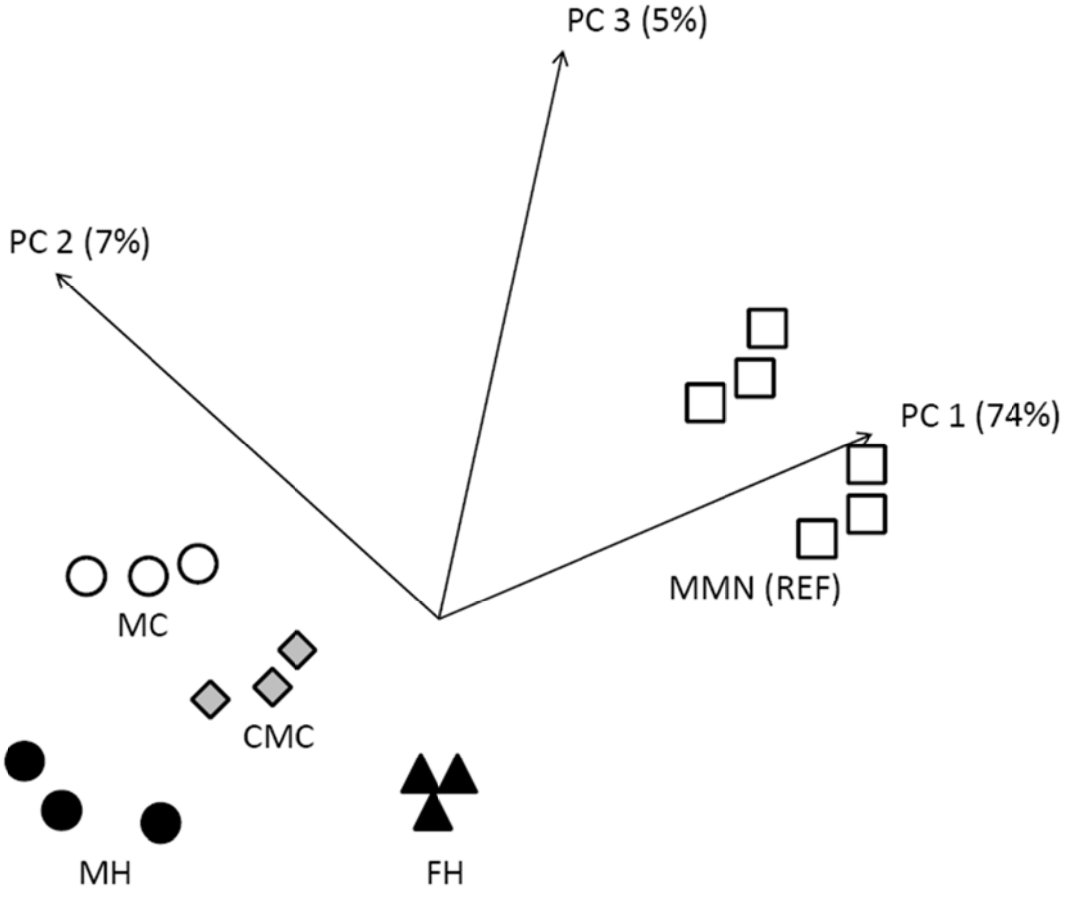

Figure S6. Comparison of the CAZymes overexpressed more than twice in a cellulose medium compared with a glucose medium for *Pa. involutus*, *Po. placenta*, and *Ph. chrysosporium*. The genes belonging to CAZyme families that were upregulated at least two folds are shown in lines; the number of gene models in each family is shown by the number of cells; and the fold increase (cellulose/glucose medium) of the corresponding gene model is given in each cell; values in bold indicate that the gene model bears a cellulose binding domain. Microarray data for *Ph. chrysosporium* and *Po.placenta* growing on microcrystalline cellulose (AVICEL) and glucose media were downloaded from the GEO database (accession numbers GSE14736 and GSE12540, respectively.

| CAZYmes | *Pa. involutus* | *Po. placenta* | *Ph. chrysosporium* |
|---|---|---|---|
| GH5 | 4.0, 2.5, 2.2 | 2.4 | **5.8**, **5.2**, **4.4**, 2.1 |
| GH61 | 2.2 | | 20.1, **5.8**, **3.4**, **2.5** |
| GH18 | 3.0 | | **2.1**, 2.0 |
| GH27 | 2.1 | 2.1 | |
| GH15 | 2.0 | 2.3 | |
| GH28 | | 2.3 | 2.7 |
| GH1 | | 5.0, 2.8 | 3.5 |
| GH10 | | 3.7 | **3.6** |
| GH2 | | 2.7, 2.3, 2.3 | |
| GH3 | | 2.3 | |
| GH6 | | | **7.4** |
| GH7 | | | 9.2, **5.7**, **4.0**, **2.8**, 2.6 |
| GH9 | 2.7 | | |
| GH11 | | | **13.4** |
| GH12 | | | 2.7, 2.3 |
| GH16 | | 4.0, 3.7, 2.3, 2.1 | |
| GH25 | | | 2.2 |
| GH30 | | 2.3 | |
| GH53 | | 3.9 | |
| GH55 | | 5.4, 3.1 | |
| GH71 | | | 2.1 |
| GH(BNR repeat) | | | 3.0, 2.9, 2.2 |
| GH(unassigned) | | 2.2 | |
| GT2 | | 2.1 | |
| CE4 | | 2.4 | |
| PL3 | 2.0 | | |
| Lysozyme | | | 2.9 |
| CBM | | | 5.6, 2.1 |

Figure S7. Proposed involvement of involutin in a hydroquinone-mediated Fenton reaction by *Pa. involutus*. Modified from Kerem *et al* (2009).

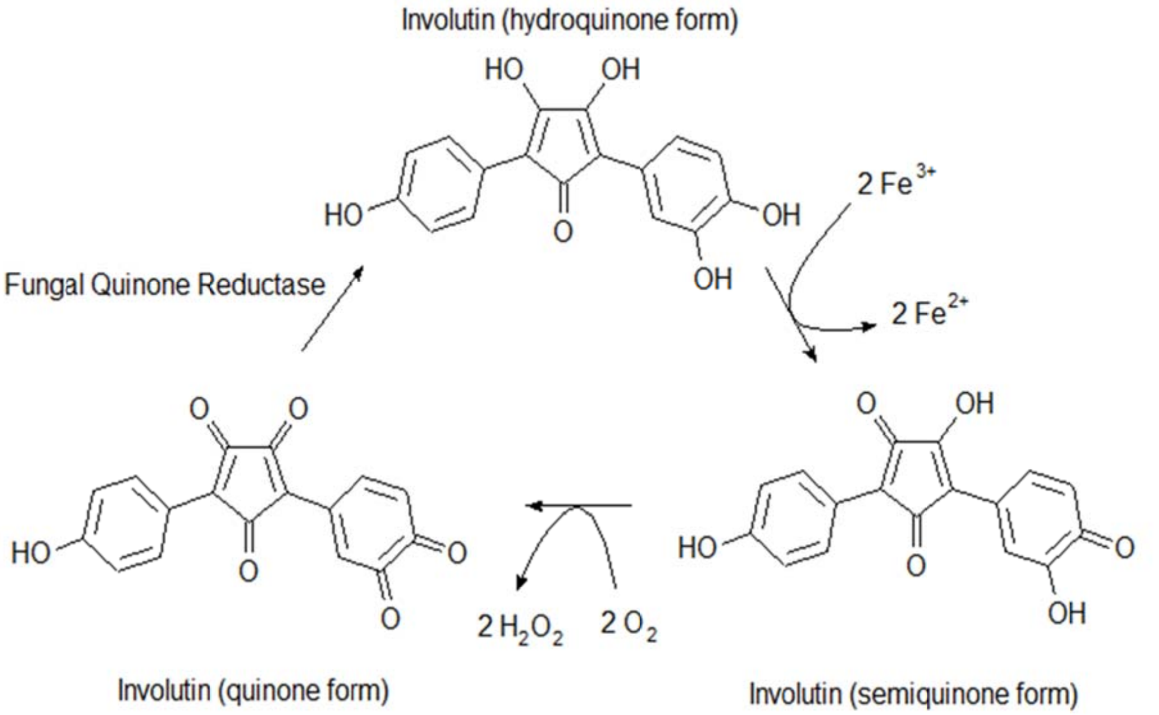

Table S1. Changes in C, N, and nutrients during the conversion of soil organic matter by *Pa. involutus*. The fungus was grown for 7 days using three different organic matter extracts as substrates; forest litter extracted with hot water (Forest Hot), and a maize compost extracted with cold (Maize Cold) or hot (Maize Hot) water, respectively. To avoid carbon limitation, glucose was added to the extract. Reference is the composition in the extracts before being added to the fungus, Inoculated is the concentration of the elements after 7 days of incubation. Abbreviations used: TOC, Total Organic Carbon; TN, Total Nitrogen; ND, Not detected. Concentrations are given in $mg.l^{-1}$. Three inoculated samples were pooled before being analyzed.

| | **Forest Hot** | | **Maize Hot** | | **Maize Cold** | |
|---|---|---|---|---|---|---|
| | Reference | Inoculated | Reference | Inoculated | Reference | Inoculated |
| TOC | 2770 | 1600 | 1225 | 520 | 1170 | 348 |
| Glucose C | 1000 | ND | 1000 | ND | 1000 | ND |
| TOC-Glucose C | 1770 | 1600 | 225 | 520 | 170 | 348 |
| TN | 100 | 47 | 164 | 109 | 230 | 121 |
| C/N | 28 | 34 | 7 | 5 | 5 | 3 |
| P | 1 | 1 | 6.7 | 6.4 | 13.6 | 11.4 |
| K | 1 | 3 | 15 | 14 | 29 | 24 |
| Ca | 3 | 2 | 8 | 6 | 12 | 9 |
| Mg | 5 | 1 | 4 | 3 | 6 | 5 |
| Fe | 0.668 | 0.575 | 0.023 | 0.019 | 0.008 | 0.006 |

Table S2. Data used for interpreting peaks in the synchronous fluorescence (SF) spectra.

| Compound | Nb of Phenol cycles | Nb of non-aromatic cycles | Fluorescence peak | Formula | Reference |
|---|---|---|---|---|---|
| Phenylalanine | 1 | 0 | 264 nm | 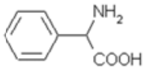 | This paper |
| Cresol | 1 | 0 | 279 nm | 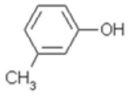 | (Ferrari and Mingazzini, 1995) |
| Tyrosine | 1 | 0 | 280 nm | 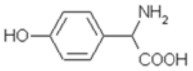 | This paper |
| Veratryl alcohol | 1 | 0 | 284 nm | 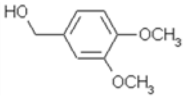 | This paper |
| Tryptophan | 1 | 1 | 294 nm | 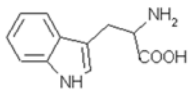 | This paper |
| Naphthol | 2 | 0 | 325 nm | 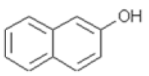 | (Ferrari and Mingazzini, 1995) |
| Naphtylamine | 2 | 0 | 358 nm | 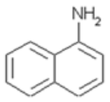 | (Ferrari and Mingazzini, 1995) |
| ABTS | 2 | 2 | 398 nm | 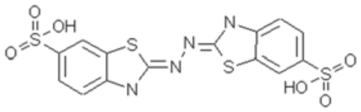 | This paper |
| Involutin | 2 | 1 | 462 nm | 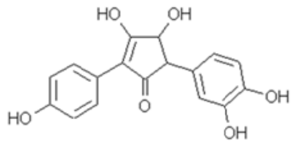 | This paper |
| Riboflavin | 3 | 0 | 472 nm | 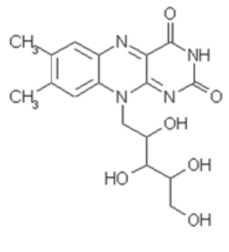 | (Ferrari and Mingazzini, 1995) |

Table S3. Putative identification of the pyrolysis compounds of the forest hot (FH) extract that were most affected by the inoculation of *Pa. involutus*. Results are presented as the ratio between average relative peak size in the 3 incubated samples (size of the peak divided by the sum of all peak sizes in the sample) and the relative peak size in the reference sample ("Fold" column; NF=Newly formed). The dominant masses of each pyrolysate are also reported.

| Nature | Compound | Dominant masses | | Fold |
|---|---|---|---|---|
| >5X decrease in peak size after inoculation | | | | |
| Phenols | (S)-5-Hydroxymethyl-2[5H]-furanone | 84 | 55 | 0 |
| Polysaccharides | 2,5-Dimethyl-4-hydroxy-3(2H)-furanone | 128 | 43 | 0.15 |
| Polysaccharides | Dianhydromannitol | 86 | 43 | 0.19 |
| 2-5x decrease in peak size after inoculation | | | | |
| Monoaromatics | 1,2-Benzenediol, 3-methyl- | 78 | 124 | 0.30 |
| Monoaromatics | Toluene | 91 | 92 | 0.41 |
| Monoaromatics | Ethylbenzene | 91 | 106 | 0.44 |
| Monoaromatics | 1,4-Benzenediol, 2-methyl- | 124 | 123 | 0.49 |
| N-compounds | Oxypurinol | 109 | 152 | 0.22 |
| N-compounds | 2-Propanamine, N-methyl-N-nitroso- | 102 | 43 | 0.24 |
| N-compounds | 2-Pyrrolidinone, 5-(ethoxymethyl)-? | 84 | 41 | 0.35 |
| N-compounds | N-methylpyrrole (1-methyl) | 80 | 81 | 0.37 |
| N-compounds | Pyridine, 4(3?)-methyl- | 93 | 66 | 0.44 |
| N-compounds | 3-methylpyridine | 66 | 93 | 0.49 |
| Polyaromatics | Indene | 115 | 116 | 0.40 |
| Polysaccharides | Furan, 2-ethyl-5-methyl- | 95 | 110 | 0.20 |
| Polysaccharides | 2,5-Furandione | 54 | 98 | 0.21 |
| Polysaccharides | Furan, 2,5-dimethyl- | 96 | 95 | 0.25 |
| Polysaccharides | 4H-Pyran-4-one, 2,3-dihydro-3,5-dihydroxy-6-methyl- | 43 | 44 | 0.28 |
| Polysaccharides | 2(3H)-Furanone, 5-heptyldihydro-? | 85 | | 0.34 |
| Polysaccharides | Cyclopentanone | 55 | 84 | 0.34 |
| Polysaccharides | Propanoic acid | 74 | 45 | 0.35 |
| Polysaccharides | 2-Cyclopenten-1-one, 3-ethyl- | 81 | 110 | 0.35 |
| Polysaccharides | (S)-(+)-2',3'-Dideoxyribonolactone | 85 | 57 | 0.41 |
| Polysaccharides | 2-Acetyl-5-methylfuran | 109 | 124 | 0.42 |
| Polysaccharides | 4H-Pyran-4-one, 2,3-dihydro-3,5-dihydroxy-6-methyl-? | 43 | 101 | 0.42 |
| Polysaccharides | 2-Methylfuran | 53 | 82 | 0.43 |
| Polysaccharides | 2-Cyclopenten-1-one, 2,3-dimethyl- | 67 | 110 | 0.44 |
| Polysaccharides | 2-Butanone | 43 | 72 | 0.46 |
| Polysaccharides | 2-propylfuran | 53 | 81 | 0.48 |
| 2-10X increased after inoculation | | | | |
| Lignins | Vanillin of iso-Vanillin of 4-Formylguaiacol | 151 | 152 | 2.17 |
| Lignins | Vanillic Acid | 153 | 168 | 2.18 |
| Phenols | Phenol, 2-butyl- | 107 | 150 | 2.70 |
| Phenols | Phenol, 2,4-bis(1,1-dimethylethyl)- | 191 | 57 | 8.44 |

| | | | | |
|---|---|---|---|---|
| Polysaccharides | (2H)-Furan-3-one | 84 | 54 | 2.19 |
| Polysaccharides | 1,4:3,6-Dianhydro-à-d-glucopyranose | 69 | 57 | 2.21 |
| Polysaccharides | 2-Propanone, 1-hydroxy-? | 43 | 74 | 2.28 |
| Polysaccharides | 2-Furancarboxaldehyde, 5-(hydroxymethyl)- | 97 | 126 | 2.32 |
| Polysaccharides | 4H-Pyran-4-one, 5-hydroxy-2-methyl- | 126 | 69 | 2.57 |
| Polysaccharides | 4-hydroxy-5,6-dihydro-(2H)-pyran-2-one | 114 | 58 | 2.64 |
| Polysaccharides | 3-Penten-2-one | 69 | 43 | 3.05 |
| Polysaccharides | Dianhydrorhamnose | 113 | 128 | 3.45 |
| Polysaccharides | Trimethylene oxide | 58 | 57 | 3.74 |
| Polysaccharides | 2-Cyclopentene-1,4-dione | 96 | 42 | 4.01 |
| Polysaccharides | 2-Butanone, 1-(acetyloxy)- | 43 | 57 | 4.18 |
| Polysaccharides | Propanoic acid, 2-oxo-, methyl ester | 43 | 102 | 4.19 |
| >10X increased after inoculation | | | | |
| N-compounds | N-Nitrosodimethylamine | 74 | 42 | 10.83 |
| Lignins | 1,2-Benzenediol, n-methyl- | 124 | 78 | NF |
| N-compounds | Thymine | 126 | 55 | NF |
| N-compounds | Xanthosine | 57 | 43 | NF |
| Phenols | Phenol, p-tert-butyl-? | 135 | 107 | NF |
| Polyaromatics | Indene-like | 187 | 202 | NF |
| Polysaccharides | 1,3-Propanediol? | 57 | 58 | NF |
| Polysaccharides | 2-Furancarboxylic acid | 112 | 95 | NF |
| Unknown | Unknown | 56 | | NF |

Table S4. Identification of secreted proteins expressed by *Pa. involutus* during the degradation of organic matter. cDNA libraries were constructed from RNA isolated from mycelium grown on forest hot (FH), maize hot (MH) and maize cold extracts (MC), respectively. Listed are 18 TAST trappant sequences encoding secretory proteins potentially involved in the degradation of lignocelluloses.

| Mechanism | | TAST contig | Acc. num ber | Annotation | Secretion[a] | Isotig[b] | Isogroup[b] | Microarray[c] |
|---|---|---|---|---|---|---|---|---|
| Polysaccharide modification | Plant cell wall degradation | TAST-1 | | Expansin | * | 981 | 3131 | 1.4 |
| | | TAST-2 | | GH9 (endo-beta-1,4-glucanase) | ** (?) | 6851 | 2174 | 2.7 |
| | | TAST-5 | | GH61 | * | 9086 | 4563 | 1.2 |
| | | TAST-6 | | GH61 | * | 6210 | 1670 | 1.4 |
| | | TAST-7 | | GH61 | * | 8715 | 4164 | 1.1 |
| | Fungal cell wall modification | TAST-16 | | GH18 (Chitinase) | * | 730 | 1442 | 2.5 |
| | | TAST-17 | | CBM19 (Chitin-binding module) | * | 1424 | 8339 | 1.3 |
| | | TAST-18 | | CBM50 (Chitin/peptidoglycan binding module) | ** | 1220 | 6201 | 1.4 |
| | Biomass degradation | TAST-3 | | GH5 (glucan 1,3-beta-glucosidase) | * | 1072 | 4550 | 1.5 |
| | | TAST-4 | | GH17 (glucan 1,3-beta-glucosidase) | ** (?) | 6313 | 1735 | 0.8 |
| | Unclear role | TAST-15 | | GH16; transglucosylase in beta-1,6-glucan synthesis | ** | 4936 | 872 | 2.4 |
| Lignin degradation | | TAST-11 | | Laccase | * | 8036 | 3437 | 3.4 |
| | | TAST-9 | | Aromatic peroxygenase | ** | 977 | 3016 | 1.2 |
| | | TAST-10 | | Cytochrome p450 oxidoreductase | * | 4020 | 455 | 1.3 |
| H2O2 production | | TAST-12 | | Galactose/glyoxal oxidase | ** (?) | 4725 | 743 | 0.7 |
| Iron reduction and homeostasis | | TAST-14 | | NADH:flavin oxidoreductase NADH oxidase | ** | 699 | 1277 | 1.0 |
| | | TAST-8 | | Short-chain dehydrogenase | ** | 796 | 1743 | 0.8 |
| Oxalate metabolism | | TAST-13 | | Oxalate decarboxylase | * | 98 | 45 | 1.5 |

[a]Prediction of secretion signals using the SignalP "*" or the Secretome "**" algorithm. "(?)" indicates that the sequences are likely to have an incomplete or comprised N-terminus; thus it is not possible to identify any secretion signal in the N-terminus.

[b]Isotig and and Isogroup refer to contigs and gene models generated by 454 sequencing. They can be retrieved from the *Pa. involutus* EST database http://mbio-serv2.mbioekol.lu.se/Paxillus/Hybrid/ (add "paxillus_" to the given isotig and isogroup numbers).

[c]Regulation as analyzed by DNA microarray analysis. The value given is the mean fold value of the transcript levels in the pairwise comparisons FH/MMN, MH/MMN, MC/MMN.

## Appendix S1

## Supplementary experimental procedures

**Preparation of organic matter extracts.** The cold water fraction for the Maize compost (MC) was extracted by shaking 120 g of material in 600 ml of MQ water for 24 h. The hot water extracts for the maize compost and forest soil (MH and FH, respectively) were obtained by boiling 120 g of material in 600 ml of MQ water for 1 h. The MC, MH and FH extracts were: 1) filtered through a nylon mesh; 2) sequentially vacuum-filtered with GF-D, GF-A and GF-F filters (Millipore, USA), respectively; 3) centrifuged 1h at 17 000 rpm at 4 $^{o}$C; 4) filtered through 0.45 µm using Millex-HV non-sterile Filter units (Millipore, USA); 5) vacuum-filtered through 0.2 µm using Stericup system (Millipore, USA) to remove particles and to sterilize the extracts. To decrease the concentration of low molecular weight compounds, the extracts were finally ultra-filtered with an Amicon Stirred Ultrafiltration Cell using regenerated cellulose membranes of a cut-off value of 10 kDa and 1 kDa, respectively (Millipore, USA). The volume of the extract was adjusted to 400 ml. The same preparations of the FH, MH and MC extracts were used in all experiments. Analysis of the chemical composition of the MH extract (total N content and synchronous fluorescence spectra) before and after being added to the glass bead plates did not reveal any detectable adsorption of macromolecules onto the glass beads.

**Chemical analysis.** Total organic carbon concentrations were measured using a TOC Analyzer (Shimadzu), and total nitrogen (TN) using the same apparatus with a TNM-1 detector. Concentrations of P, K, Ca, Mg and Fe were measured using ICP-AES (Perkin-Elmer Optima 3000 DV). Glucose concentration was measured using the glucose (GO) assay kit provided by Sigma-Aldrich (Germany).

Samples for Fourier transform infrared (FTIR) spectroscopy were prepared by drying (vacuum over night at 4 $^{o}$C) 5 ml of the organic matter extracts. One volume of powder was then ground and homogenized with three volumes of KBr in a mortar. The FTIR spectrum of the sample-KBr mixture was recorded using a Bruker IFS66 v/s spectrometer under mild vacuum (ca. 4 mbar)

and at 25.0 ± 0.2 ºC. Data were collected in diffuse reflectance mode using a praying mantis diffuse reflectance attachment (Harrick Sci.). Each spectrum was the result of 1000 consecutive scans at a resolution of 4 $cm^{-1}$. Pure and ground KBr was used as background.

Synchronous fluorescence spectra were obtained using a Perkin-Elmer LS50B fluorescence spectrophotometer. Samples (750 µl) were kept at room temperature (20 °C) and processed at a 10 nm bandwidth and 25 nm offset (Δλ=25 nm) between excitation and emission. Samples were contained in a 1ml quartz cuvette with a 1 cm path length. The sample spectra were recorded at different dilutions in MQ water (1, 1/5, 1/10, 1/20, 1/40, 1/100) because fluorescence peaks of SF-detectable compounds have different sensitivities to dilution (Tuan, 1982). A fifty times dilution was chosen as it gave the best signal/noise ratio and as its optical density was low enough to interfere with fluorescence spectra (Senesi *et al.,* 1991). Assignments of these regions were based on previously published data (Artz *et al.*, 2008; Stevenson and Goh, 1971; Senesi *et al.*, 2003).

Pyrolysis gas chromatography-mass spectrometry (Py-GC/MS) was conducted to analyze the detailed changes in chemical composition occurring during the degradation of the FH extract. A volume of 5 ml of FH reference sample and 3 ml FH inoculated samples were vacuum dried overnight at 4° C. Samples were then analyzed by Py-GC/MS (Perkin Elmer TurboMass/ Autosystem XL with Frontier Lab double Shot pyrolyser). Each (GC/MS) peak was attributed to a pyrolysis product (molecule, i.e vanillic acid), and, when possible, the broad molecular origin (lignin, phenolic compounds, aromatic compounds, polyaromatics, N-containing compounds and polysaccharides) of each product was inferred according to literature (Table S2). Then, for each sample, we calculated the sum (S) of the peak areas (A) of the pyrolysis products of the same broad molecular origin (for example: $S_{Lignin}=A_{Lignin\ compound\ 1}+A_{Lignin\ compound\ 2}+\ldots+A_{Lignin\ compound\ n}$), and normalized it by the total peak areas in the sample $N_{Lignin}=S_{Lignin}/(S_{phenoliccompounds}+S_{Aromatics}+S_{Polyaromatics}+S_{N\text{-}containingcompounds}+S_{Polysaccharides})$. To compare reference (n=1) and inoculated (n=5) samples, we calculated the ratio (R) of this normalized value in the incubated samples compared with the reference one ($R_{Lignin}=N_{Lignin}^{Incubated}/\ N_{Lignin}^{Reference}$). However, this ratio compares only relative amounts of compounds. To evaluate enrichment or

loss in a class of compounds, we compared this ratio to the loss in total organic carbon measured in the samples ("No change level").

Size-exclusion chromatography was performed using a HiLoad™ 16/60 Superdex™200 column (GE Healthcare). Twenty replicates of the inoculated FH, MH and MC samples were pooled and aliquot of 150 µl was applied to the column. The buffer used was 0.100 M Tris-HCl (pH 7.2) and 0.15 M NaCl with a flow rate of $1 ml.min^{-1}$. Elution was followed at 280 nm. Fractions of 8 ml were collected and assayed using synchronous fluorescence spectroscopy and for reducing sugars using the phenol-sulphuric acid method (Dubois *et al.,* 1956). Blue Dextran (2000 kDa), Ferritin (440 kDa), Catalase (232 kDa) and BSA (67 kDa) were used as molecular size markers.

**Enzyme activity measurements.** Preliminary experiments showed that the organic matter extracts (FH, MH and MC) contained compounds that significantly interfered with the measurements of extracellular enzyme activities. To remove such compounds including phenolic compounds, the extracts were treated with PVPP (PolyVinyl Poly Pyrrolidone) (modified from Pierpoint, 1996), followed by acetone precipitation. In brief, a volume of 250 µl of PVPP solution (3.2 %, in MQ water) was added to 1ml of the FH, MH and MC extracts. The mixture was vortexed (4 times, 15 sec), centrifuged at 13000 rpm for 20 min (4 $^{o}$C), and the pellet containing PVPP and adsorbed phenolic compounds was discarded. The PVPP treatment was repeated four times. Proteins in the supernatant were precipitated by adding four volumes of ice-cold acetone. The mixture was stored overnight at -30$^{o}$C, and precipitated proteins were recovered by centrifugation at 4500 rpm for 20 min (4 $^{o}$C). The pellet was finally dissolved in 1ml of MQ water.

Six extracellular enzyme activities were measured that could be involved in the degradation of lignocellulose: laccases, lignin peroxidases, other oxidases, cellobiohydrolases, glucuronidases and xylanases. Aliquots of 100 µl of the PVPP/acetone purified samples were analyzed in a total volume of 1 ml, adjusted with a 0.1 M citrate buffer. Laccase activity was measured by recording increase of absorbance at 525 nm of 0.5 M syringaldazine, at pH 4.0, for a reaction time of 20 min at 20 $^{o}$C (Leonowicz and Grzywnowicz, 1981). Lignin peroxidase activity was measured by

recording increase of absorbance at 310 nm of 0.4 mM veratryl alcohol, at pH 3.0, with 0.4 mM of $H_2O_2$, for a reaction time of 2 min at 20$^o$C (Tien and Kirk, 1988). Overall oxidase activity was measured by recording increase of absorbance at 420 nm of 2 mM ABTS, at pH 4, for a reaction time of 20 min at 20$^o$C (Palmieri *et al.,* 1997). All the absorbance measurements were recorded with an Ultrospec 3000 UV/visible spectrophotometer (Pharmacia Biotech). Cellobiohydrolase activity was measured by recording increase of fluorescence at 460 nm (for an excitation wavelength of 355 nm) of 0.4mM Methylumbelliferyl-β-D-Cellobioside, at pH 4.0, for a reaction time of 40 min at 20 $^o$C (Courty *et al.,* 2005). Glucuronidase activity was measured by recording increase of fluorescence at 460 nm (for en excitation wavelength of 355 nm) of 0.4 mM Methylumbelliferyl-β-D-Glucuronide hydrate, at pH 4.0, for a reaction time of 40 min at 20$^o$C (Courty *et al.,* 2005). Xylanase activity was measured by recording increase of absorbance at 595 nm of 5.75g.l$^{-1}$ of RBB-Xylan, at pH 5.0, for a reaction time of 30 min at 30 $^o$C (Biely *et al.,* 1985). Extracellular protease activity was measured at pH 3.0, according to Twining (Twining, 1984) using FITC-BSA as a synthetic substrate (FITC-BSA: 2 mg.ml$^{-1}$, citrate buffer 0.1 M, 200 µl of purified enzyme-containing sample, 410 µl total volume).The reaction was incubated overnight at 37 $^o$C. The substrate which did not react was precipitated using 200 µl of 10% TCA, stored for 1hour in the dark, centrifuged at 13000 rpm for 5 min, and 40 µl of the supernatant was mixed with 710 µl of 0.4 M Na-Borate Buffer pH 8.0. The fluorescence activity was read at 490 nm excitation and 525 nm emission. For all enzyme measurements, the absorbance or fluorescence units measured were corrected by time; blanks (containing water instead of sample) were subtracted, and the difference with the reference sample (inoculated-reference) was calculated. The number of nmol of substrate liberated by the enzyme was calculated using extinction molar coefficients for syringaldazine (ɜ=65000mol$^{-1}$.cm$^{-1}$), veratryl alcohol (ɜ=9300mol$^{-1}$.cm$^{-1}$), ABTS (ɜ=36000mol$^{-1}$.cm$^{-1}$), RBB (ɜ=6170mol$^{-1}$.cm$^{-1}$), Para Nitrophenyl (ɜ=1730mol$^{-1}$.cm$^{-1}$), FITC (ɜ=60000mol$^{-1}$.cm$^{-1}$) and using standard curve for methylumbelliferone. The fluorescence activity was measured in a Perkin-Elmer LS50B fluorescence spectrophotometer.

**Iron-reducing compounds.** The capacity of *Pa. involutus* to produce iron-reducing compounds when grown on organic matter extracts was examined using a ferrozine assay (Goodell *et al.,*

2006). A 100 µl aliquot of the FH, MH and MC extracts was mixed with 10 µl of 15 mM ferrozine and 10 µl of 1.2mM $FeCl_3$ hexahydrate. The absorbance at 562 nm (formation of the $Fe^{2+}$-ferrozine complex) was recorded after 2 min of incubation (v<50% vmax). The total amount of $Fe^{3+}$ in the mixture at the beginning of the reaction was 12 nmol in a volume of 120 µl.

**RNA extractions.** Fungal mycelia from each treatment and each biological replicate were quickly collected from the bead plates and directly dropped into a clean mortar filled with liquid $N_2$ and homogenized using a pestle. The resulting powder was collected into 50-ml Falcon tubes and stored at -80°C until use. Total RNA was isolated from each sample using the RNeasy Plant Mini Kit (Qiagen) according to the manufacturer's instructions, and using the RLC buffer. Total RNA was eluted in either 60 or 100 µl of $H_2O$ and stored at -20°C until use. For quality and concentration assessments all samples were investigated using a 2100 Bioanalyzer and a RNA 6000 Nano kits (Agilent).

**Construction and screening of TAST cDNA library.** Equal amounts of total RNA isolated from mycelium grown in the MC, MH and FH media were combined into one sample. Poly(A) RNA isolation, cDNA synthesis, and Transposon Assisted Signal Trapping (TAST) cDNA library construction were essentially carried out as described previously (Grell *et al.,* 2011), except that two parallel ligations were set up with different vector/cDNA ratios: one tenth of the cDNA preparation was cloned into 15 and 45 ng, respectively, of the vector pMHas7i (kindly provided by K. Schnorr, Novozymes) using one unit T4 DNA ligase from the SMART cDNA Library Construction Kit (Clontech). The cDNA library was transposon-tagged, transformed into *E. coli* cells, and signal-trapped clones were selected (Becker *et al.,* 2004; Grell *et al.,* 2011). Colonies surviving the section were cultivated in 96-well growth blocks. In total, 576 clones were sequenced. Sequencing and assembly of DNA sequences was performed with the sequencing primers seqBv2 (5'-TACGTGCCGATCAACGTCTCA-3') and seq-Av2 (5'-GGGGCGAAAACTCTCAAGGA-3'), an ABI 3730 XL automatic DNA sequencer using BigDye v. 3.1 terminators and the PhredPhrap package (Ewing *et al.,* 1998).

The sequences were assembled into 348 contigs. The contig sequences were annotated using the Blast2GO tool (Götz et al., 2008), and information in the PFAM database (Finn *et al.,* 2006) and the CAZy database (Cantarel *et al.,* 2009). For a predicted glycoside hydrolase (GH) 16 family protein, the subfamily was predicted using the structured GH16 glycoside hydrolase database (Strohmeier *et al.,* 2004). Signal peptide-triggered secretion and non-classical secretions signals were identified using the SignalP 3.0 and SecretomeP algorithms (Emanuelsson *et al.,* 2007). TAST-sequences with missing or compromised N-terminus were filled-in using sequence information from the corresponding isotig in the *Pa. involutus* EST database. Matches between TAST-sequences and isotigs were performed using the Blastn tool (Altschul *et al.,* 1990).

**Sequencing of cDNA libraries.** Two cDNA libraries were constructed for EST (expressed sequence tag) sequencing. The first library was made from RNA isolated from mycelium grown in the MMN medium. The second library was constructed by combining equal amounts of RNA extracted from mycelium grown for 7 days on the MH, MC and FH, respectively. After total RNA purification (see above), mRNA was isolated from each sample using approximately 100 μg and the Absolutely mRNA Purification Kit (Agilent). The purified mRNA was then used as starting material in the cDNA Library Preparation protocol (GS FLX Titanium Series) provided by 454/Roche and followed by sequencing on one full PicoTiter plate (PTP) of sequencing for each library.

Assembly of EST sequences was performed in three steps. First, ESTs generated in earlier experiments using the Sanger method (Johansson *et al.,* 2004; Morel *et al.,* 2005) were assembled. Second, the EST sequences from the two 454 libraries were assembled. Third, the two sets of consensus sequences were assembled. The assembly result was a set of contig sequences representing various splice variants (isotigs in Roche vocabulary).

In step 1, the 21,163 collected Sanger sequences had an average length of 713 nucleotides; 15 of the reads contained rRNA sequences and were removed. The remaining sequences were assembled by using the Mira assembler, version 3.0.3 (Chevreux *et al.,* 2004) with default parameters. The result was an assembly of 2,298 contigs (isotigs) representing 13,015 sequences.

The remaining sequences were trashed mainly due to low quality scores. In step 2, an 454 assembly made with the gsAssembler software (Newbler) (Margulies *et al.,* 2005) was kindly provided by the Department of Energy, Joint Genome Institute. The assembly contained 13,641 isotigs with sequence lengths >50 nucleotides. These isotigs had a mean length of 1,736 nucleotides and represented 2,114,871 reads.

In step 3, consensus sequences from the Sanger and 454 assemblies were assembled by using the CAP3 assembler (Huang and Madan, 1999). This resulted in an assembly of 13,654 isotigs. All read sequences were then mapped against the consensus sequences of the isotigs with the aid of the ssaha2 software (Ning *et al.,* 2001) using default settings. Probably due to a higher stringency in the ssaha2 algorithm as compared to the assembly programs, there were 781 isotigs that no reads mapped to. These isotigs represented 98,281 reads. After removal of these isotigs and reads, the final numbers in the assembly was 12,873 isotigs representing 2,029,605 reads. The average isotig length was 1,563 nucleotides. The vast majority of reads derive from the 454 sequencing (>99.9%). Based on the gsAssembler output, the 12,873 isotigs mapped to 8,620 genes (isogroups in Roche vocabulary). EST sequences are available at GenBank SRA046093.

**Bioinformatic analyses of EST sequences.** An EST database was constructed based on the assembly data that may be accessed from http://mbio-serv2.mbioekol.lu.se/Paxillus/Hybrid/. The database allows access to UniProt annotations, BLAST output, PFAM domains (Finn et al., 2006) and Gene Ontology (GO) (Ashburner *et al.,* 2000). Similarity searches against UniProt (Apweiler *et al.,* 2004) database were performed for each isotig consensus sequence. With an e-value threshold of 1e-10, 10,178 of the isotigs showed similarity with sequences from UniProt. In the database, isotig annotations are inferred from the best Blastx hit.

To get isotigs encoding for proteins potentially involved in the degradation of lignocellulose, we compiled information on such proteins being identified from in the genome, transcriptome and secretome analysis of the wood decaying brown-rot fungus *Po. placenta* and the white-rot fungus *Ph. chrysosporium* (Martinez *et al.*, 2004; Martinez *et al.*, 2009; Vanden Wymelenberg *et al.*, 2010; Vanden Wymelenberg *et al.*, 2006; Kersten and Cullen, 2007) and other species

(Vaillancourt *et al.*, 2006; Gilbert, 2010; Hofrichter *et al.*, 2010).The analysis generated a list of 123 "target proteins" involved in polysaccharide or lignin degradation, iron metabolism, oxalic acid metabolism and $H_2O_2$ production. Identification of putative homologs of these sequences among the *Pa. involutus* isotigs was done in two steps. During step 1, the names of the 123 target proteins were used as queries to search for related sequences among the annotated *Pa. involutus* isotigs. The search generated 212 putative homologs. During step 2, a more extensive search for homologs was done by retrieving a broad range of query sequences related to those of the 123 "target proteins". First, 1,981 sequences having the names of the 123 "target proteins" in their annotations were obtained from the UniProt database of fungal proteins. Second, 3,198 sequences displaying a high similarity to those of the 123 target proteins were retrieved from the UniProt database of fungal proteins by blastp searches. The two sets of sequences were compiled to a list of 5,179 protein sequences. Using this list as a query, a tblastn search (E-value threshold=$1e^{-10}$) against *Pa. involutus* EST database generated 556 putative homologs among the isotigs. The putative *Pa. involutus* homologs identified during the 2 steps were manually annotated. Carbohydrate-active enzymes were annotated based on PFAM searches and similarity to such enzymes in the UniProt and GenBank databases, and they were classified using the CAZyme nomenclature (Cantarel *et al.,* 2009). The resulting list contained 266 *Pa. involutus* isotigs encoding for proteins potentially involved in the degradation of lignocellulose. Prediction of the presence secretory signal peptides and non-classical secretion signals were done using the SignalP 3.0 and SecretomeP algorithms, respectively (Emanuelsson *et al.,* 2007).

**Microarray experiments.** From a combined assembly of the two cDNA libraries the 12,873 isotigs were identified and were used for the construction of 12-plex 135K oligonucleotide microarray (Nimblegen/Roche). Of those 12,214 isotigs could be used for probe/reporter construction and allowed up to 10 probes per isotig in tiled design. The microarray analyses were performed as single-label hybridizations. For each hybridization and each sample 10 μg of total RNA was used for cDNA synthesis using the SuperScript Double-Stranded cDNA Synthesis Kit (Invitrogen) according to the manufacturer. For quality assessments the produced cDNA was analyzed on a 2100 Bioanalyzer and DNA 7500 kit (Agilent). For sample labeling the One-Color DNA Labeling Kit (NimbleGen/Roche) was used according to the manufacturer. After labeling,

each sample received a Sample Tracking Control (NimbleGen/Roche) and hybridizations were immediately performed in a Hybridization System 4 (NimbleGen/Roche) for at least 16 h and according to the manufacturer. The following washing procedure was accordingly (NimbleGen/Roche) and the slides were finally scanned in an Agilent High-Resolution Microarray Scanner set at 2 μm. The raw images were bursted and processed using the NimbleScan software v. 2.5 according to the manufacturer (NimbleGen/Roche). Normalized (log(2)-transformed) values were brought into the Omics Explorer ver. 2.2 (Qlucore) for PCA and statistical analyses. Hybridization signal were obtained for 12,214 isotigs.

## References

Altschul,S.F., Gish,W., Miller,W., Myers,E.W., Lipman,D.J. (1990) Basic local alignment search tool. *J Mol Biol* **215:** 403-410.

Apweiler,R., Bairoch, A.,Wu, C.H., Barker,W.C., Boeckmann,B., Ferro S. et al. (2004) UniProt: the Universal Protein knowledgebase. *Nucleic Acids Res* **32:** D115-D119.

Artz,R.R.E., Chapman,J.S., Robertson,A.H.J., Potts,J.M., Laggoun-Défarge,F., Gogo,S. et al. (2008) FTIR spectroscopy can be used as a screening tool for organic matter quality in regenerating cuover petalands. *Soil Biol Biochem* **40:** 515-527.

Ashburner,M., Ball,C.A., Blake,J.A.,.Botstein,D., Butler,H., Cherry,J.M. et al. (2000) Gene ontology: tool for the unification of biology. The Gene Ontology Consortium. *Nat Genet* **25:** 25-29.

Becker,F., Schnorr,K., Wilting,R., Tolstrup,N., Bendtsen,J.D. and Olsen PB. (2004**).** Development of in vitro transposon assisted signal sequence trapping and its use in screening *Bacillus halodurans* C125 and *Sulfolobus solfataricus* P2 gene libraries. *J Microbiol Methods* **57:** 123-133.

Biely,P., Mislovicova,D. and Toman,R. (1985) Soluble chromogenic substrates for the assay of endo-1,4-beta-xylanases and endo-1,4-beta-glucanases. *Anal Biochem* **144:** 142-146.

Cantarel,B.L., Coutinho,P.M., Rancurel,C., Bernard,T., Lombard,V. and Henrissat,B. (2009) The Carbohydrate-Active EnZymes database (CAZy): an expert resource for Glycogenomics. *Nucleic Acids Res* **37:** D233-D238.

Chevreux,B., Pfisterer,T., Drescher,B., Driesel,A.J., Muller,W.E., Wetter,T. and Suhai,S. (2004) Using the miraEST assembler for reliable and automated mRNA transcript assembly and SNP detection in sequenced ESTs. *Genome Res* **14:** 1147-1159.

Courty,P.-E., Pritsch,K., Schloter,M., Hartmann,A. andGarbaye,J. (2005) Activity profiling of ectomycorrhiza communities in two forest soils using multiple enzymatic tests. *New Phytol* **167:** 309-319.

Dubois,M., Gilles,K.A., Hamilton,J.K., Rebers,P.A. and Smith, F. (1956) Colorimetric method for determination of sugars and related compounds. *Anal Chem* **28:** 350-356.

Emanuelsson,O., Brunak,S., von Heijne,G. and Nielsen,H. (2007) Locating proteins in the cell using TargetP, SignalP and related tools. *Nat Protoc* **2:** 953-971.

Ewing,B., Hillier,L., Wendl,M.C. and Green,P. (1998) Base-calling of automated sequencer traces using *Phred*. I. Accuracy assessment. *Genome Res* **8:** 175-185.

Ferrari,G.M. and Mingazzini,M. (1995) Synchronous fluorescence spectra of dissolved organic matter (DOM) of algal origin in marine coastal waters. *Mar Ecol Prog Ser* **125:** 305-315.

Finn,R.D., Mistry,J., Schuster-Bockler,B., Griffiths-Jones,S., Hollich,V., Lassmann,T. et al. (2006) Pfam: clans, web tools and services. *Nucleic Acids Res* **34:** D247-D251.

Gilbert,H.J. (2010) The biochemistry and structural biology of plant cell wall deconstruction. *Plant Physiol* **153:** 444-455.

Goodell,B., Daniel,G., Jellison,J., and Qian,Y. (2006) Iron-reducing capacity of low-molecular weight compounds produced in wood by fungi. *Holzforschung* **60:** 630-636.

Götz,S., Garcia-Gomez,J.M., Terol,J., Williams,T.D., Nagaraj,S.H., Nueda,M.J. et al. (2008) High-throughput functional annotation and data mining with the Blast2GO suite. *Nucleic Acids Res* **36:** 3420-3435.

Grell,M.N., Jensen,A.B., Olsen,P.B., Eilenberg,J., and Lange,L. (2011) Secretome of fungus-infected aphids documents high pathogen activity and weak host response. *Fungal Genet Biol* **48:** 343-352.

Hofrichter,M., Ullrich,R., Pecyna,M.J., Liers,C. and Lundell,T. (2010) New and classic families of secreted fungal heme peroxidases. *Appl Microbiol Biotechnol* **87:** 871-897.

Huang,X. and Madan,A. (1999) CAP3: A DNA sequence assembly program. *Genome Res* **9:** 868-877.

Johansson,T., Le Quéré,A., Ahrén,D., Söderström,B., Erlandsson,R., Lundeberg,J., Uhlén,M. and Tunlid,A. (2004) Transcriptional responses of *Paxillus involutus* and *Betula pendula* during formation of ectomycorrhizal root tissue. *Mol Plant Microbe Interact* **17:** 202-215.

Kacurakova M, Capek P, Sasinkova V, Wellner N, Ebringerova A. (2000)**.** FTIR study of plant cell wall model compounds: pectic polysaccharides and hemicelluloses. Carbohyd Polym 43**:**195-203.

Kerem,Z., Jensen,K.A., and Hammel,K.E. (1999) Biodegradative mechanism of the brown rot basidiomycete *Gloeophyllum trabeum*: evidence for an extracellular hydroquinone-driven fenton reaction. *FEBS Letters* **446:** 49-54.

Kersten,P. and Cullen,D. (2007) Extracellular oxidative systems of the lignin-degrading Basidiomycete *Phanerochaete chrysosporium*. *Fungal Genet Biol* **44:** 77-87.

Leonowicz,A. and Grzywnowicz,K. (1981) Quantitative estimation of laccase forms in some white-rot fungi using syringaldazine as a substrate. *Enzyme Microb Technol* **3:** 55-58.

Margulies,M., Egholm,M., Altman,W.E., Attiya,S., Bader,J.S., Bemben,L.A. et al. (2005) Genome sequencing in microfabricated high-density picolitre reactors. *Nature* **437:** 376-380.

Martinez,D., Challacombe,J., Morgenstern,I., Hibbett,D., Schmoll,M., Kubicek,C.P. et al. (2009) Genome, transcriptome, and secretome analysis of wood decay fungus *Postia placenta* supports unique mechanisms of lignocellulose conversion. *Proc Natl Acad Sci U S A* **106:** 1954-1959.

Martinez,D., Larrondo,L.F., Putnam,N., Gelpke,M.D., Huang,K., Chapman,J. et al. (2004) Genome sequence of the lignocellulose degrading fungus *Phanerochaete chrysosporium* strain RP78. *Nat Biotechnol* **22:** 695-700.

Morel,M., Jacob,C., Kohler,A., Johansson,T., Martin,F., Chalot,M. and Brun, A. (2005) Identification of genes differentially expressed in extraradical mycelium and ectomycorrhizal roots during *Paxillus involutus-Betula pendula* ectomycorrhizal symbiosis. *Appl Environ Microbiol* **71:** 382-391.

Ning,Z., Cox,A.J. and Mullikin,J.C. (2001) SSAHA: a fast search method for large DNA databases. *Genome Res* **11:** 1725-1729.

Palmieri,G., Giardina,P., Bianco,C., Scaloni,A., Capasso,A. and Sannia,G. (1997) A novel white laccase from *Pleurotus ostreatus*. *J Biol Chem* **272:** 31301-31307.

Pierpoint,W.S. (1996) The extraction of enzymes from plant tissues rich in phenolic compounds. *Methods Mol Biol* **59:** 69-80.

Senesi,N., D'Orazio,V. and Ricca,G. (2003) Humic acids in the first generation of EUROSOILS. *Geoderma* **3:** 325-344.

Senesi,N., Miano,T.M., Provenzano,M.R., and Brunetti,G. (1991) Characterization, differentiation, and classification of humic substances by fluorescence spectroscopy. *Soil Science* **152:** 259-271.

Stevenson,F.J. and Goh,K.M. (1971) Infrared spectra of humic acids and related substances. *Geochimica et Cosmochimica Acta* **35:** 471-483.

Strohmeier,M., Hrmova,M., Fischer,M., Harvey,A.J., Fincher,G.B. and Pleiss,J. (2004) Molecular modeling of family GH16 glycoside hydrolases: potential roles for xyloglucan transglucosylases/hydrolases in cell wall modification in the poaceae. *Protein Sci* **13:** 3200-3213.

Tien,M. and Kirk,T.K. (1988) Lignin peroxidase of *Phanerochaete chrysosporium*. *Methods Enzymol* **161:** 238-249.

Tuan,V.-D. (1982) Synchronous luminescence spectroscopy: methodology and applicability. *Appl Spectroscopy* **36:** 576-581.

Twining,S.S. (1984) Fluorescein isothiocyanate-labeled casein assay for proteolytic enzymes. *Anal Biochem* **143:** 30-34.

Vaillancourt,F.H., Bolin,J.T. and Eltis,L.D. (2006) The ins and outs of ring-cleaving dioxygenases. *Crit Rev Biochem Mol Biol* **41:** 241-267.

Vanden Wymelenberg,A., Gaskell,J., Mozuch,M., Sabat,G., Ralph,J., Skyba,O. et al. (2010) Comparative transcriptome and secretome analysis of wood decay fungi *Postia placenta* and *Phanerochaete chrysosporium*. *Appl Environ Microbiol* **76:** 3599-3610.

Vanden Wymelenberg,A., Minges,P., Sabat,G., Martinez,D., Aerts,A., Salamov,A. et al. (2006) Computational analysis of the *Phanerochaete chrysosporium* v2.0 genome database and mass spectrometry identification of peptides in ligninolytic cultures reveal complex mixtures of secreted proteins. *Fungal Genet Biol* **43:** 343-356.
